# Glioma-derived LRIG3 interacts with NETO2 in tumor-associated macrophages to modulate microenvironment and suppress tumor growth

**DOI:** 10.1038/s41419-023-05555-z

**Published:** 2023-01-13

**Authors:** Youwei Li, Wei Wang, Xiaoshuang Hou, Wenda Huang, Po Zhang, Yue He, Baofeng Wang, Qiuhong Duan, Feng Mao, Dongsheng Guo

**Affiliations:** 1 Department of Neurosurgery, Tongji Hospital, Tongji Medical College, Huazhong University of Science and Technology, Wuhan, Hubei People’s Republic of China; 2 Department of Biochemistry and Molecular Biology, Tongji Medical College, Huazhong University of Science and Technology, Wuhan, Hubei People’s Republic of China

**Keywords:** CNS cancer, Tumour immunology, Cancer microenvironment

## Abstract

Tumor-associated macrophages (TAMs) account for 30–50% of glioma microenvironment. The interaction between glioma tumor cells and TAMs can promote tumor progression, but the intrinsic mechanisms remain unclear. Herein, we reported that soluble LRIG3 (sLRIG3) derived from glioma tumor cells can block the M2 polarization of TAMs via interacting with NETO2, thus suppressing GBM malignant progression. The expression or activity of ADAM17 in glioma cells was positively correlated with the expression of sLRIG3 in cell supernatant. Soluble LRIG3 can suppress the M2-like polarity transformation of TAMs and inhibit the growth of tumor. High expression of LRIG3 predicts a good prognosis in patients with glioma. Mass spectrometry and Co-immunoprecipitation showed that sLRIG3 interacts with the CUB1 domain of NETO2 in TAMs. Silencing or knockout of NETO2 could block the effect of sLRIG3, which inhibited the M2-like polarity transformation of TAMs and promoted GBM tumor growth. However, overexpressing His-target NETO2 with CUB1 deletion mutation does not fully recover the suppressive effects of sLRIG3 on the TAM M2-polarization in NETO2-Knockout TAMs. Our study revealed vital molecular crosstalk between GBM tumor cells and TAMs. Glioma cells mediated the M2 polarization of TAM through the sLRIG3-NETO2 pathway and inhibited the progression of GBM, suggesting that sLRIG3-NETO2 may be a potential target for GBM treatment.

## Introduction

Glioblastoma (GBM) is one of the most aggressive and lethal primary malignant tumors of the adult central nervous system. Despite the multi-mode treatment containing surgery, radiotherapy, chemotherapy, electric field therapy, targeted drug therapy, and supportive care in recent years, the median overall survival of GBM patients is still less than two years, and the 5-year survival rate is limited to 10% [1]. The tumor microenvironment (TME), which contains tumor cells, stromal cells, infiltrating immune cells, corresponding secretory factors, extracellular matrix, and vessels [2], plays a crucial role in supporting malignant growth and progression of GBM [3, 4]. Tumor-associated macrophages (TAMs) are the largest non-tumor cells in glioma microenvironment, accounting for 30–50% of cells in tumor and play an important role in the progression of glioma [5, 6]. According to the activation state of cells, TAMs can be divided into tumor-suppressing M1-like subgroup and tumor-promoting M2-like subgroup. Among them, M1-like TAMs secrete pro-inflammatory cytokines, phagocytose cancer cells to present microbial antigen to activate the adaptive immune system [7, 8]. Conversely, M2-like TAMs secrete anti-inflammatory cytokines and display immune-suppressive function to promote GBM malignant behavior and tumor growth [9, 10]. It has been reported that the density and M1–M2 polarity transformation of TAMs are related to the prognosis of glioma patients [11]. Targeting TAMs can modulate the malignant progression of GBM in mice cell line-derived xenograft model [11–13], suggesting that TAM is a potential therapeutic target for improving the treatment of GBM. Although TAMs play a crucial role in GBM malignancies, the molecular mechanism remains to be elucidated.

GBM cells in TME secrete soluble proteins, like CCN1 [14], LOX [15], and Periostin [11], which play significant roles in maintaining the M2 phase phenotype of TAM in TME to promote tumor growth. For example, osteopontin derived from GBM cells recruits and maintains M2-like macrophage phenotype by mediating its major receptor Integrin αvβ5, leading to tumor invasion, proliferation, and angiogenesis [16]. Our recent study indicated that LRIG3 can suppress the growth of GBM cell lines and tumor xenografts in Cell line-derived xenograft model by downregulating the activity of MET/phosphatidylinositol 3-kinase/Akt signaling pathway, thus playing an anti-tumor role in glioma [17]. Soluble LRIG3 (sLRIG3) has recently been identified in the cystic fluid of cystic glioma, the serum of glioma patients, and supernatant of glioma cell lines. ELISA assay indicated that the serum sLRIG3 protein level was positively correlated with overall survival time of patients with high-grade glioma [17]. However, how sLRIG3 derived from glioma cells mediates GBM propagation through TME has not been defined.

To interrogate the effect of LRIG3 on TME of GBM, we first utilized The Cancer Genome Atlas (TCGA) database to verify the relationship between LRIG3 expression and stromal and immune signatures or immune cell types of GBM TME. We showed that low LRIG3 expression was correlated with significant enrichment of both M1-like and M2-like macrophages, while high LRIG3 expression predicted increased CD8+ effector T cells. Consistently, we identified that sLRIG3 in GBM microenvironment can attenuate the tilt towards an M2-dominant TAM population and suppress tumor growth, suggesting that sLRIG3 may mediate GBM biology by targeting TAMs. Our recent study identified that A disintegrin and metalloprotease 17 (ADAM17), also called TACE (tumor necrosis factor-α-converting enzyme), can regulate the release of soluble LRIG1 [18], one of the members of LRIG gene family [19]. Herein, we find that LRIG3 is preferentially expressed by GBM tumor cells compared with TAMs in TME. Silencing ADAM17 expression in GBM tumor cells was correlated with low level of sLRIG3 protein in cellular supernatant, which indicating that ADAM17 may modulate the release of sLRIG3. Moreover, we screened out the interaction protein Neuropilin and Tolloid-like protein 2 (NETO2) of sLRIG3 through the mass spectrometry assay. The effect of sLRIG3 on attenuating the tilt towards an M2-dominant TAM population was partly blocked by knocking down NETO2 in TAMs. NETO2 has been found to be aberrantly expressed in various malignant tumors [20–22], but the specific mechanism of NETO2 in tumor progression remains unclear. Herein, we explored the significance of sLRIG3-NETO2 signaling in regulating TAM polarization and provide novel insight into how this signaling remodels TAMs and tumor microenvironment. We identified sLRIG3-NETO2 signaling axis as a key molecule link between glioma cells and TAMs to modulate GBM malignant development.

## Materials and methods

### Human glioma specimens

Human glioma surgical specimens were obtained from the Department of Neurosurgery, Tongji Hospital, Huazhong University of Science and Technology (Wuhan, China), with the informed consents from the patients or their guardians, according to a protocol approved by the Research Ethics Committee of Tongji Hospital, Tongji Medical College, Huazhong University of Science and Technology. At least two nerve pathologists participated in the histopathological diagnosis of glioma specimens based on world Health Organization (WHO) classification. The pathological features of the glioma specimens were shown in Supplementary Table 1. All procedures were performed according to the standard of the Helsinki Declaration and approved by the institutional ethics committees of Tongji Medical College of Huazhong University of Science and Technology.

### Cell culture and induction

The human glioma cell line U118MG, U87MG and human embryonic renal cell line HEK293T were purchased from American Type Culture Collection. The GBM cell line Fu is the primary glioma cell line constructed by our laboratory. Mouse glioma cell line GL261 and mouse monocyte-macrophage cell line RAW264.7 were donated by Prof. Feng Zhu, Tongji Medical College. All cell lines tested negative for mycoplasma contamination. The U118MG, U87MG, HEK293T, FU, GL261 and RAW264.7 cells were cultured with DMEM containing 10% fetal bovine serum (FBS) at 37 °C in a humidified 5% CO_2_ incubat or. All cell lines were tested and authenticated in Wuhan pricella Co., Ltd. by short tandem repeat profiling.

Mouse bone marrow-derived macrophages (BMDMs) were isolated from the femoral bone marrow of anesthetized mice. After carefully removing the femoral muscle with scissors and break the femoral bone at the joint, the bone marrow was washed with serum-free RPMI 1640 medium. After centrifugation, the marrow derived cells were cultured in RPMI 1640 medium containing MCSF (50 ng/mL) and 10% FBS for 3 days at 37 °C in a humidified 5% CO_2_ incubator. Fresh media was replaced on day 3.

For obtaining RAW264.7/BMDM-derived TAMs, RAW264.7 or BMDM were cultured in sLRIG3(+) or sLRIG3(−) medium from GL261 cell line for 48 h.

### Cell transfection

The cell transfection assay was conducted using Simple-Fect reagent (Wuhan Signal Dawn Biotechnology LTD) according to the manufacturer’s instructions. Corresponding blank vectors were added for adjustment to ensure that the total amount of DNA transfected in each group was equal. Stable cell lines were screened with Puromycin and G418. In addition, the sequences for shRNAs in this study were summarized in Supplementary Table 2.

### sLRIG3(+) and sLRIG3(−) medium

The glioma cell lines overexpressing FLAG-LRIG3 were cultured for 48 h and then the conditional medium (CM) was filtered with a 0.45 μM Millex filter (Millipore Corporation, USA) and collected. The CM was incubated with blank protein A/G or protein A/G pre-combined with FLAG antibody for 3 h. Liquids was collected after centrifugation. sLRIG3(−) or sLRIG3(+) conditioned medium for subsequent experiments were obtained by mixing existing conditional media with fresh 10% FBS 1640 medium in a ratio of 1:2.

### Knockout Using CRISPR

We selected the conserved sequence of mouse *Neto2* DNA sequence and designed 4 sets of sgRNA sequences (Supplementary Table 2) using ZhangFeng’s laboratory design tool [23]. The 4 target sgRNA sequences were cloned into LentiCRISPRv2 plasmid backbone and transfected into cells. Stable cell lines were screeneded with puromycin, and monoclonal cells were selected to obtain NETO2-knockout cell lines.

### ELISA

Cell culture medium supernatant was collected and filtered by a 0.45 μM Millex filter (Millipore Corporation, USA). Protein levels of LRIG3 (R&D, DY3495), IL10 (Beyotime, PI522) and iNOS (NOVUS, NBP2-80256) were determined via corresponding ELISA kit under protocols.

### Quantitative real-time PCR

CFX96 Real-time PCR system (Bio-RAD) was utilized for qRT-PCR detection. Primer sequences used in this study are shown in Supplementary Table 3.

### Isolation of nuclear protein

Samples were collected and the nuclear proteins for each sample were extracted using Nuclear Protein Extraction Kit (Solarbio, R0050).

### Western bloting and immunoprecipitation (IP)

Cells for Western bloting or IP were lysed with biyuntian cell lysis buffer. For the cells used for WB, ultrasound was performed at 20% power for 30 s on ice, repeated for 3 times, followed by centrifugation, and loading buffer was added into the obtained supernatant. The obtained samples were utilized for further study. For IP samples, 5% power ultrasound was performed on ice for 30 s, repeated for three times, and the supernatant was obtained after centrifugation. 500 mg of each protein sample was taken and incubated with corresponding antibody and protein A/G (Santa Cruz Biotechnology, Inc.) at 4 °C for 12 h in a shaker. Samples were washed with PBS containing 0.1% Triton-100 for 4 times. The obtained samples were utilized for further study. Primary antibodies for Western bloting are listed here: Goat anti-LRIG3 (1:1000, R&D, AF3495), Rabbit anti-Tubulin (1:1000, Proteintech, 10068-1-AP), Rabbit anti-ADAM17 (1:1000, ABclonal, A0821), Mouse anti-CD86 (1:1000, ABclonal, A1199), Mouse anti-ARG1 (1:500, Santa Cruz, sc-271430), Mouse anti-FLAG (1:2000, Sigma-Aldrich, F1804), Rabbit anti-His (1:500, Proteintech, 10001-0-AP), Mouse anti-His (1:3000, Proteintech, 66005-1-Ig), Rabbit anti-β-Actin (1:1000, Proteintech, 20536-1-AP), Rabbit anti-NETO2 (1:2000, Sigma-Aldrich, HPA013180), Mouse anti-P65 (1:1000, Proteintech, 66535-1-Ig), Rabbit anti-Phospho-NF-κB p65 (Ser536) (1:1000, Cell Signaling, 3033 S), Rabbit anti-Histone-H3 (1:5000, Proteintech, 17168-1-AP), Mouse anti-CCR2 (1:500, ABclonal, A2855), Mouse anti-CCR4 (1:1000, Abacm, ab216560), Mouse anti-ITGB3 (1:1000, ABclonal, A19073).

### Pull down and mass spectrometry

After cultured in sLRIG3(+) medium for 48H, TAMs were collected and lysed with biyuntian cell lysis buffer. These samples were centrifuged with 5% power ultrasound on ice and the supernatant was obtained. The supernatants were co-incubated with protein A/G agarose containing anti-FLAG for 12 h. After centrifugation, samples were washed with 0.1% Triton-100 PBS four times. The obtained samples were subsequently analyzed using Thermo’s Q X Active Plus liquid mass spectrometry system.

### Preparation of single cell suspensions of brain tumor

Brain tumor of tumor-bearing mice were cut into small pieces (2–3 mm diameter) and then enzymatically dissociated by collagenase I (150U/ml, BasalMedia) in 1640 media supplemented with 2% FBS and DNAse (50 ng/ml, Worthington) for 40 min at 37 °C. After being filtered with a 70-micron filter, RBC Lysis Solution was used to eliminate erythrocytes in samples. The single-cell suspensions obtained from the above process were used for subsequent flow cytometry assay.

### Flow cytometry analyses

samples were seeded in 6 cm dish and induced according to the experimental protocol. Pre-prepared cells were further incubated with the corresponding fluorochrome-labelled primary antibody at 25 °c for 30 min. Sony ID7000 Spectral Cell Analyzer was used for flow cytometry analyses. Data were analysed and presented using FlowJo 10.8.1. Antibodies for flow cytometry are listed here: FITC anti-mouse/human CD11b (BioLegend, 101206, clone: M1/70), PE anti-mouse CD206 (BioLegend, 141705, clone: C068C2), BB515 anti-mouse CD8 (BD Pharmingen, 564422), PE anti-mouse CD86 (BD Pharmingen, 553692), PERCP anti-mouse/human CD11b (BD Pharmingen, 566416), APC anti-mouse CD206 (BD Pharmingen, 565250), APC-CY7 anti-mouse FSV780 (BD Pharmingen, 565388), BV421 anti-mouse CD3 (BD Pharmingen, 562600), BV510 anti-mouse CD45 (BD Pharmingen, 563891), BV421 anti-mouse F4/80 (BD Pharmingen, 565411), FITC AnnexinV (BD Pharmingen, 556420) and Fixable Viability Stain 780 (BD Pharmingen, 565388).

### Transwell migration assay

2 × 10^4^ mouse monocyte-macrophage cell line RAW264.7 or 1 × 10^5^ primary mouse BMDMs were re-suspended in DMEM/RPMI 1640 medium of 10% FBS and seeded in transwell upper chamber of 24-well plates (8.0 µm Pore) with conditioned medium in the lower chamber. After 8H (RAW264,7) or 24H (mouse BMDMs), the cells were fixed and stained with crystal violet (0.05%, sigma). The number of cells in each field was detected under microscope.

### Immunofluorescent staining

TAMs induced by conditioned medium were seeded onto cover glass (sigma, USA) followed by fixing in 4% paraformaldehyde for 20 min, permeating in triton-x100 for 30 min, and incubating at room temperature with 10%FBS for 30 min. Then the samples were washed and incubatd with primary antibody in wet box at 4 °c overnight. On day 2, cells were washed and incubated with Alexa Fluor 488 (green for NETO2) or Alexa Fluor 546 (red for LRIG3) secondary antibody at room temperature in wet box for 2 h. Chromatin was stained by DAPI. The images were observed and collected under the fluorescence microscope.

### Immunohistochemistry

The immunohistochemical tissues of human and mouse were obtained from patients of the Department of Neurosurgery, Tongji Hospital, Huazhong University of Science and Technology (Wuhan, China) and mouse CDA model. Immunohistochemistry (IHC) primary antibody used in this study includes Goat anti-LRIG3 (1:50, R&D, AF3495), Rabbit anti-CD163 (1:100, Abcam, ab182422), Rabbit anti-IBA1 (1:2000, Abcam, ab178846), Rabbit anti-NETO2 (1:200, Sigma-Aldrich, HPA013180), Mouse anti-ARG1 (1:200, Santa Cruz, sc-271430), Rabbit anti-CD8 alpha (1:500, Abcam, ab209775) Rabbit anti-BAX (1:300, Boster, BA0412), and Rabbit anti-BCL2 (1:300, Boster, BA0315-1). The IOD scores of LRIG3, CD163, IBA1, ARG1 or CD8A in human GBM or mouse CDA models were quantified by software imageJ under ×400 magnified microscope with an Olympus Imaging System Microscope in the whole regions of each tumor specimen.

### RNA sequencing

The induced primary BMDMs were seeded in conditioned medium. Pre-prepared cells further were fully lysed with Trizol and sent to Wuhan Boyuezhihe Biotechnology LTD for transcriptome Sequencing. Illunima HiSeqTM2000/ Miseq was used for sequencing and Hisat2 software was used for subsequent analysis.

### Orthotopic allografts model

The mice from the same batch were randomized into different groups and used for orthotopic allografts model. GBM cells were implanted into the right frontal lobe of the intracranial mouse to construct the orthotopic mouse animal model. To directly investigate tumor growth, GL261 mouse glioma cell lines expressing pLVX-puro-Luciferase were used to establish mice model. To simulate the tumor immune microenvironment, 6-week-old male C57BL/6 mice with normal immune system were selected. To determine the role of the NETO2 expression of TAMs in regulating TME and tumor progression, TAMs expressing shNETO2 or shMock (5 × 10^4^) and GL261-LUC expressing LRIG3 (1.5 × 10^5^) were co-implanted into mouse brain. To determine the role of the NETO2 CUB1 domain in promoting glioma progression, TAMs expressing Mock, NETO2-FL or NETO2-Del1 were co-implanted into the mouse brain with GL261-LUC expressed LRIG3 (1.5 × 10^5^). The image of tumor growth was monitored and quantified by in vivo imaging system (IVIS) Living Image software and mice were killed when neurological signs occurred. All animal experiments were approved by the Animal Ethics Committee of Tongji Hospital affiliated to Huazhong University of Science and Technology.

### Computational analysis

To analyze human GBM data, we downloaded gene expression and survival data of TCGA GBM data from cBioPortal [24]. Stromal and immune scores for each patient can be downloaded from ESTIMATE: http://bioinformatics.Mdandersond.org/ESTIMATE/ [25]. We analyzed the association between interstitial/immune score and survival. Based on the genetic background of each patient, we analyzed the correlation of LRIG3 expression level with immune-related signaling pathway, matrix/immune score, and TME cell composition.

### Statistical analyses

Unless otherwise stated, all statistical analyses for the comparison were performed using unpaired *t* test or one-way analysis of variance (ANOVA). Bar graphs were presented as means ± s.d., with statistical significance at *P < 0.05, **P < 0.01, ***P < 0.001. Bivariate correlation analysis (Pearson’s *r* test) was used to examine the correlation of two variables. LRIG3, NETO2, and macrophage markers/signatures were analyzed using GBM TCGA database and the results of immunohistochemical staining (IHC). Log-rank test (GraphPad Prism 9) was used to analyze the survival data, with statistical significance at *P < 0.05, **P < 0.01, ***P < 0.001.

## Results

### LRIG3 expression in human GBM is negatively correlated with M2-like TAMs

**Fig. 1:**
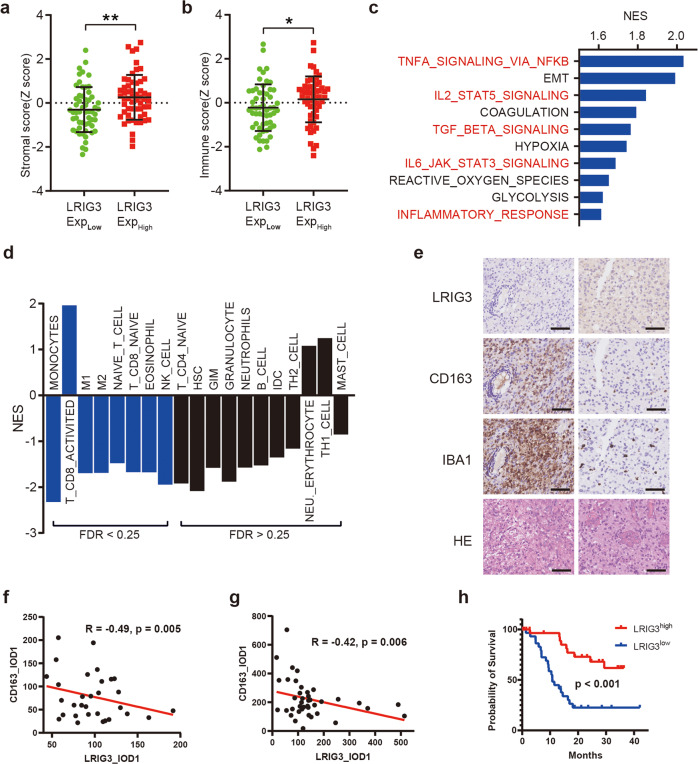
LRIG3 expression is negatively correlated with tumor-supportive TAMs in human GBM and informs good prognosis of GBM patients. **a**, **b** The stroma score (**a**) and immune score (**b**) of LRIG3-High-expression and LRIG3-low-expression patients in the TCGA GBM database. The stroma and immune scores were determined based on expression data (Yoshihara et al., 2013). Unpaired student’s *t* test. **c** GSEA analysis based on KEGG gene sets and TCGA GBM database. **d** GSEA analysis for various types of immune cells in LRIG3-High-expression and LRIG3-low-expression patients in TCGA GBM database. **e** Representative images of HE staining and the low- and high-expression levels of LRIG3, CD163, and IBA1 in human GBM tissue microarrays. Scale bar, 50 mm. **f**, **g** Correlation analysis between LRIG3 and CD163 expression in TMA of low grade (grade 2 and 3) glioma (*n* = 31) (**f**) and GBM (*n* = 40) (**g**). Pearson’s correlation test. **h** Kaplan–Meier survival curves of LRIG3-High-expression and LRIG3-Low-expression patients in TCGA GBM database.

LRIG3 has been identified to negatively regulate multiple tyrosine kinase receptor signaling pathways thus supressing glioma growth. We previously detected sLRIG3 in serum and cystic fluid of patients with cystic glioma [17]. Soluble LRIG3 might regulate the progression of glioma through the interaction between cells in the glioma microenvironment. To evaluate the effect of sLRIG3 on glioma microenvironment, patients of TCGA GBM cohort were divided into LRIG3-high group and LRIG3-low group based on the median value of LRIG3 sequencing value. We identifed that LRIG3-high group has higher interstitial and immune signals compard with LRIG3-low group based on TCGA GBM database (Fig. 1a, b). Further GSEA analysis showed that the activity of immune-related pathways such as NF-kB, JAK-STAT3 and TGF-β was upregulated in the LRIG3-high group compared with LRIG3-low group (Fig. 1c). To identify specific immune cells associated with LRIG3 expression in GBM, we examined 19 types of immune cells in TCGA GBM using a validated gene set [24]. Bioinformatics analysis based onalgorithm Gene set enrichment analysis [26] showed that in LRIG3-high group of TCGA GBM cohort, M1/ M2-like tumor-associated macrophages were significantly decreased, while CD8+ T cells were increased (Fig. 1d).

Consistent with the bioinformatic analyses, IHC assay dmonstrated that the expression of LRIG3 was negatively correlated with the M2-like TAM marker CD163 in clinical tissue specimens (Fig. 1e–g). Meanwhile, GBM with high LRIG3 expression predicted a better prognosis (Fig. 1h). In conclusion, these findings indicated that high expression of LRIG3 was negatively correlated with enrichment of TAMs in GBM TME and predicted a great prognosis of GBM patients.

### ADAM17 promotes the secretion of soluble LRIG3 by glioma cells

**Fig. 2:**
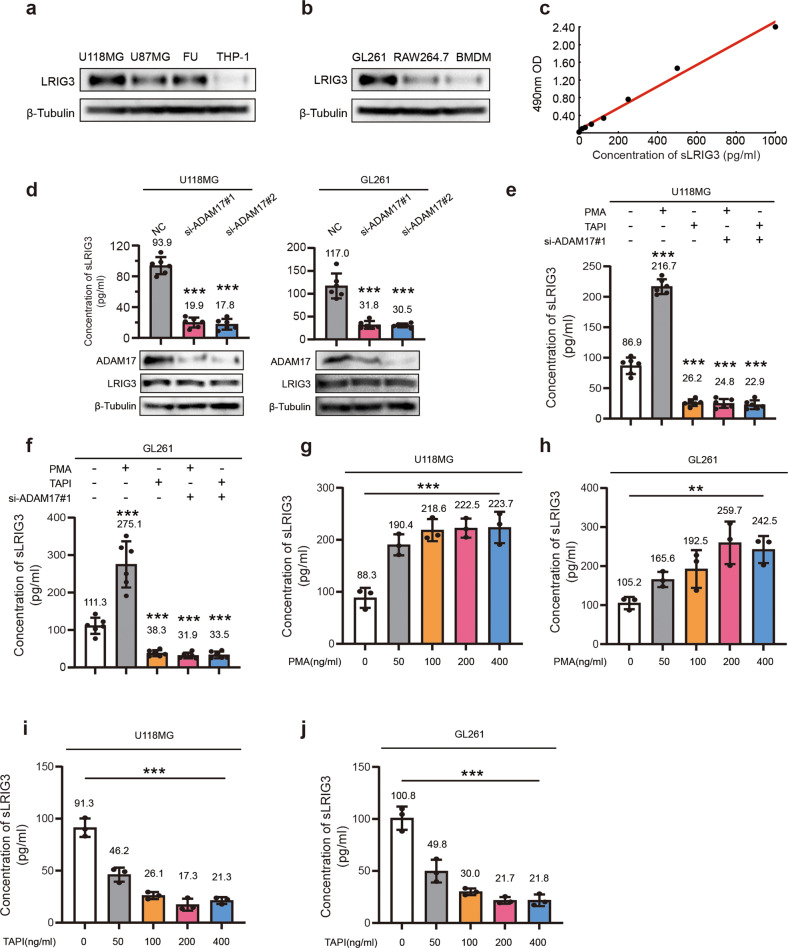
ADAM17 promotes the secretion of soluble LRIG3 by glioma cells. **a** Immunoblots for LRIG3 in the cell lysates of huamn glioma cell lines (U118MG, U87MG, FU) and macrophage-like cell line (THP-1). **b** Immunoblots for LRIG3 in the cell lysates of mouse glioma cell line (GL261) and macrophage-like cell line (RAW264.7 and BMDM). **c** ELISA standard curve showing the correlation relationship of LRIG3 concentrations and the log of the optical density 490. **d** ELISA and immunoblots for the expression of soluble LRIG3 protein of supernatant medium (up panel), ADAM17, and LRIG3 in the cell lysates (down panel) in negative control and si-ADAM17 U118MG and GL261 cells. *n* = 6 biological replicates for ELISA assay. One-way ANOVA with bonferroni correction. **e**, **f** ELISA for the expression of soluble LRIG3 protein of supernatant medium in U118MG (**e**) and GL261 (**f**) cells with stimulation of ADAM17 agonist PMA, ADAM17 inhibitor TAPI and ADAM17 siRNAs. *n* = 6 biological replicates. One-way ANOVA with bonferroni correction. **g**, **h** ELISA for the expression of soluble LRIG3 protein of supernatant medium in U118MG (**g**) and GL261 (**h**) cells in the absence or presence of increasing doses of ADAM17 agonist PMA. *n* = 3 biological replicates. One-way ANOVA. **i**, **j** ELISA for the expression of soluble LRIG3 protein of supernatant medium in U118MG (**i**) and GL261 (**j**) cells in the absence or presence of increasing doses of ADAM17 inhibitor TAPI. *n* = 3 biological replicates. One-way ANOVA.

We first detected the protein expression level of LRIG3 in GBM tumor cells and TAMs. The results of western bloting showed that LRIG3 was preferentially expressed by tumor cell line rather then TAMs in huamn/mouse cell lines (Fig. 2a, b). Recent studies reported that ADAM17 hydrolyzed LRIG1 (the first discovered gene in LRIG family) to soluble LRIG1. To explore whether ADAM17 can hydrolyze LRIG3 to sLRIG3, we silenced ADAM17 expression in U118MG, GL261 and LRIG3-overexpression GL261 (Supplementary Fig. 1a) and detected sLRIG3 expression in supernatant. Compared with the control group, the protein level of supernatant sLRIG3 in ADAM17-knockdown groups were significantly decreased (Fig. 2d). However, upregulating ADAM17 or using ADAM17 agonist PMA significantly increased the protein level of sLRIG3 in supernatant of glioma cell lines (Fig. 2e, f and Supplementary Fig. 1b). In contrast, inhibition of ADAM17 activity with the ADAM17 inhibitor TAPI reduced sLRIG3 expression in the supernatant (Fig. 2e, f and Supplementary Fig. 1b). Meanwhile, within a certain range, the dose of PMA or TAPI was significantly correlated with the expression of sLRIG3 in the supernatant (Fig. 2g–j and Supplementary Fig. 1c, d). The above evidence proved that the expression level and activity of ADAM17 in glioma tumor cells were positively correlated with the expression level of sLRIG3 in supernatant.

### Soluble LRIG3 inhibits the M2 polarization of TAMs to suppress GBM progression

**Fig. 3:**
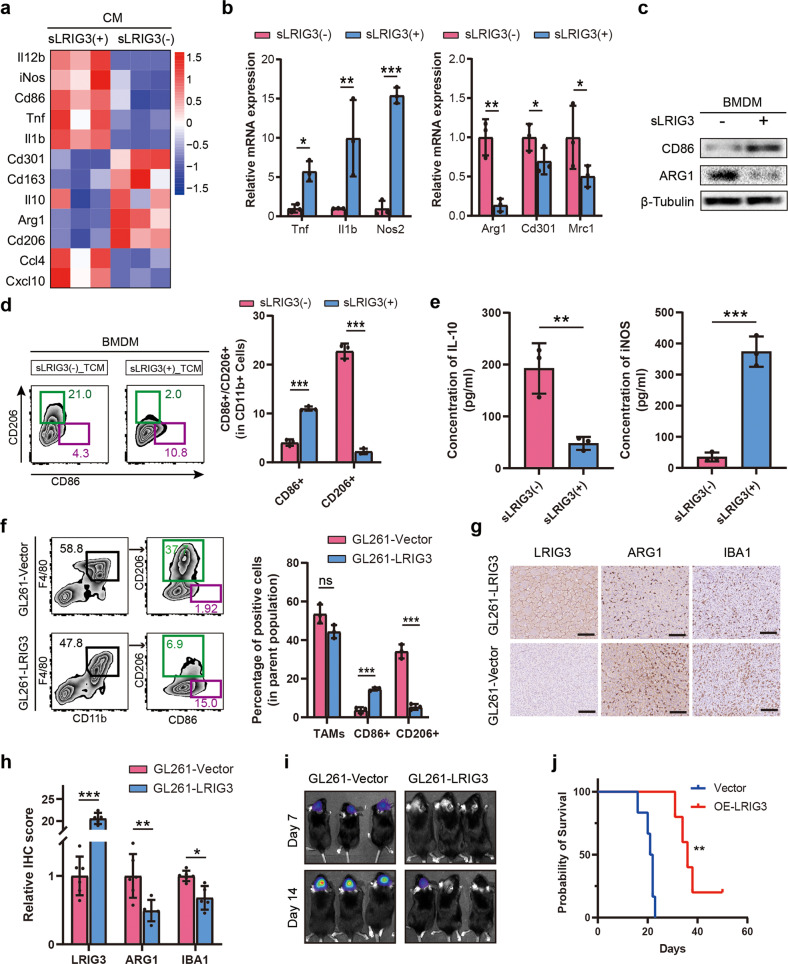
Soluble LRIG3 inhibits the M2 polarization of TAMs to suppress GBM progression. **a** Expression heatmap of the TAM M1/M2 marker genes in BMDM-derived TAMs treated with sLRIG3(+) or sLRIG3(−) CM. Data are shown as a heatmap using R software pheatmap package. Heatmap colour ranging from minimum (blue) to maximum (red) represents the relative gene expression level of BMDM-derived TAMs. **b** qRT-PCR for genes encoding TAM M1 (Left)/M2 (Right) marker proteins in BMDM-derived TAMs treated with sLRIG3(+) or sLRIG3(−) CM. Values are expressed as relative expression levels to housekeeping gene GAPDH. *n* = 3 biological replicates. Unpaired student’s *t* test. **c** Immunoblots for the expression of TAM M1 marker CD86 and M2 marker ARG1 in BMDM-derived TAMs treated with sLRIG3(+) or sLRIG3(−) CM. **d** Left panel, representative images of flow cytometry for CD86+ TAMs and CD206+ TAMs in BMDM-derived TAMs treated with sLRIG3(+) or sLRIG3(−) CM. Right panel, quantification of CD86+ and CD206+ TAMs in BMDM-derived TAMs treated with sLRIG3(+) or sLRIG3(−) CM. *n* = 3 biological replicates. Unpaired student’s *t* test. **e** ELISA for the expression of IL-10 (left panel) and iNOS (right panel) protein in the supernatant medium of BMDM-derived TAMs treated with sLRIG3(+) or sLRIG3(−) CM. *n* = 3 biological replicates. Unpaired student’s *t* test. **f** Left panel, representative images of flow cytometry for the percentage of TAMs (CD45 + CD11b + F4/80+) in leukocytes (CD45+) and the percentage of CD86+ or CD206+ TAMs in TAMs in tumor-bearing (GL261-Vector and GL261-LRIG3) C57BL/6 mice; right panel, quantification of TAMs in leukocytes and CD86+ and CD206+ TAMs in TAMs in tumor-bearing (GL261-Vector and GL261-LRIG3) C57BL/6 mice. *n* = 3 biological replicates. Unpaired student’s *t* test. **g** Immunohistochemistry (IHC) for LRIG3, ARG1, and IBA1 in tumor-bearing (GL261-Vector and GL261-LRIG3) C57BL/6 mice. Scale bar, 50 mm. **h** Quantification of Immunohistochemistry (IHC) score for LRIG3, ARG1, and IBA1 in tumor-bearing (GL261-Vector (*n* = 6/group) and GL261-LRIG3 (*n* = 5/group)) C57BL/6 mice. Unpaired student’s *t* test. **i** Representative luciferase images of three mice per group 7 and 14 days after implantation. GL261-luciferase cells (1.5 × 10^5^ cells) were transduced with blank Vector or LRIG3 plasmids. Color scale for GL261 cells: Min = 2.0e5; Max = 5.0e7. **j** Kaplan–Meier survival curves of C57BL/6 mice implanted with GL261 cells (1.5 × 10^5^ cells) transduced with blank Vector or LRIG3 plasmids. (GL261-Vector (*n* = 6/group) and GL261-LRIG3 (*n* = 5/group)). Log-rank test.

To validate the effects of sLRIG3 on TAMs in the TME, RNA sequencing was performed to analyze TAMs treated with sLRIG3(+) or sLRIG3(−) supernatant. The results showed that TAMs treated with sLRIG3(+) supernatant had the increased signature of M1-like TAM markers (*Il12b*, *iNos*, *Cd86*, *Tnf*, *Il1b*), while the signature of M2-like markers (*Cd301*, *Cd163*, *Il10*, *Arg1*, *Cd206*) was downregulated compared with the control group (Fig. 3a). Consistent with sequencing results, qPCR results showed that M2-like markers (*Cd301*, *Cd163*, *Il10*, *Arg1*, *Cd206*) were downregulated in TAMs with sLRIG3(+) treatment, while the signature of M1-like markers (*Tnf*, *Il1b*, *Nos2*) increased (Fig. 3b and Supplementary Fig. 2a). Western bloting (Fig. 3c and Supplementary Fig. 2b) and flow cytometry of cell lines (Fig. 3d and Supplementary Fig. 2c) and tumor single cell suspension (Fig. 3f) demonstrated that sLRIG3 suppressed the signature of M2-like markers and was associated with high signature M1-like markers in TAMs. Moreover, ELISA results (Fig. 3e and Supplementary Fig. 2d) showed that the concentration of iNOS (which often represents anti-tumor, pro-inflammatory M1-like macrophages) increased and IL10 (which often represents pro-tumor, anti-inflammatory M2-like macrophages) decreased in the supernatant of TAMs. Besides, transwell migration assays showed that sLRIG3(+) medium supressed the capability of TAM migration (Supplementary Fig. 2e, f). Further, the results of Western bloting (Supplementary Fig. 2g) showed that migrating-related protein receptor Integrin subunit beta 3, not CCR2 and CCR4, was downregulated in sLRIG3(+) group compared with the sLRIG3(−) group. In conclusion, the evidence above proves that sLRIG3 can inhibit the transformation of TAMs into M2-like type in the glioma microenvironment. Considering that sLRIG3 can block TAM into M2-like TAM in vitro, we suggest that sLRIG3 might play a vital role in TME, thus affecting the progression of glioma.

GL261 expressing LRIG3 and luciferase were implanted into the brains of C57BL/6 mice. Tumor size was detected by spectrum IVIS, and tumor growth was monitored at day 7 and day 14. Bioluminescence imaging showed that the growth of GL261 cell-derived allografts overexpressing LRIG3 was significantly supressed compared to the control group (Fig. 3i). Consistently, overexpressing LRIG3 in GL261 cell-derived allografts predicted better survival than blank group (Fig. 3j). Immunostaining assay demonstrated that LRIG3 expression was significantly increased in LRIG3 group compared with the control group, while M2-like marker ARG1 and total TAM marker IBA1 were downregulated (Fig. 3g, h). As well, the signature of CD8+ T cells marker CD8A increased in the LRIG3 group (Supplementary Fig. 5a, b). Collectively, these data suggest that sLRIG3 secreted by gliomas plays a vital role on inhibiting the M2-like polarity transformation of TAM and affecting the growth of gliomas.

### Discovery of sLRIG3-interacting protein NETO2 in TAMs of GBM

**Fig. 4:**
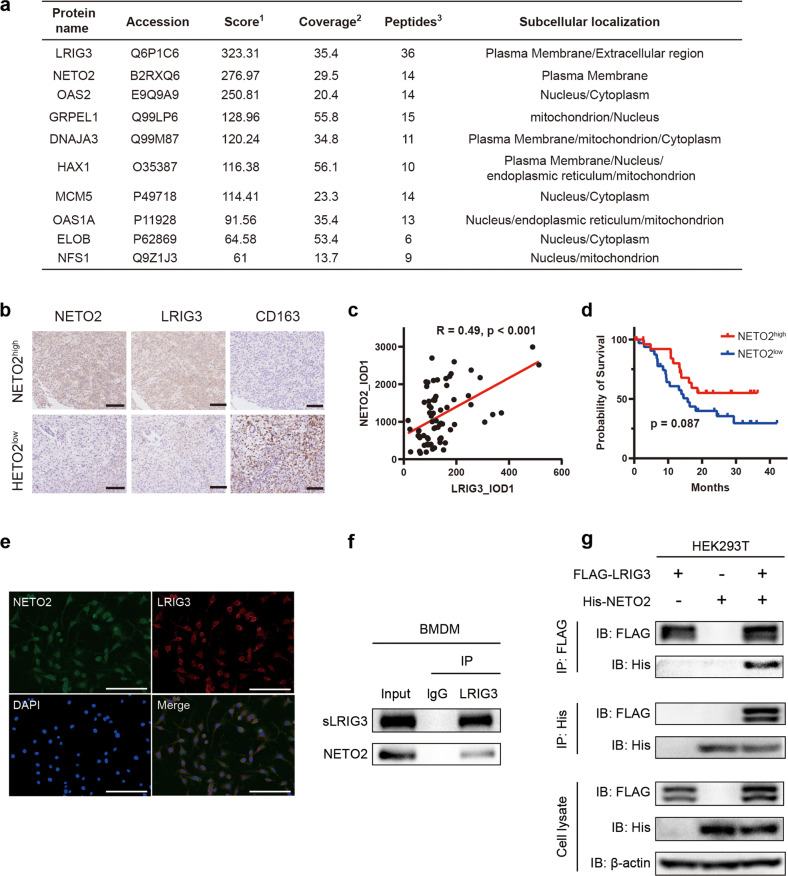
Discovery of sLRIG3-interacting protein NETO2 in TAMs of GBM. **a** Top candidates of soluble LRIG3 interacting proteins identified by pull-down and mass spectrometry analysis. ^**1**^Score: Protein scores, calculated by Proteome Discoverer application from a list of peptides identified for a particular protein, indicate the relevance of a protein. ^**2**^Coverage: Coverage of identified high-confidence peptides match the protein. ^**3**^Peptides: Number of high-confidence peptides which match the protein. **b** Representative images of the low- and high-expression levels of NETO2, LRIG3, and CD163 in human GBM tissue microarrays. Scale bar, 50 μm. **c** Correlation analysis between LRIG3 and NETO2 expression in TMA (*n* = 35) of GBM. Pearson’s correlation test. **d** Kaplan–Meier survival curves of NETO2-High-expression and NETO2-Low-expression patients in TCGA GBM database. Log-rank test. **e** Representative immunofluorescence for NETO2 (green) and LRIG3 (red) in BMDM-derived TAMs. Scale bar, 50 μm. **f** Co-immunoprecipitation of NETO2 with the LRIG3-specific antibody from cell lysates of BMDM-derived TAMs treated with sLRIG3(+) CM. Precipitation with normal mouse IgG was used as a negative control. **g** Co-immunoprecipitation of FLAG-LRIG3 and His-NETO2 from cell lysates of HEK293T transduced with pLVX-FLAG-LRIG3 and pcDNA3.1-His-NETO2. Precipitation with normal mouse IgG was used as a negative control.

To identify proteins in TAMs that may interact with sLRIG3, pull down and mass spectrometry were performed. sLRIG3(+) medium or GST sLRIG3(−) medium was co-incubated with lysate of RAW264.7-derived TAMs for 24 h, followed by co-incubating with protein A/G agarose containing anti-FLAG for 12 h. Mass spectrometry was performed to identify potential proteins interacting with the extracellular segment of LRIG3. By comparing the multiple fold change of differences among protein groups, a total of 67 differential proteins were screened out, which were considered as potential interaction proteins of sLRIG3(Fig. 4a and Supplementary Table 4). Go and KEGG enrichment functional analysis indicated that these sLRIG3-interacting proteins exerts significant biologic function in the process of cytoplasmic translation and protein folding etc. (Supplementary Table 5). The 67 proteins had scores ranging from 5.88 to 323.31 and NETO2 ranked first among these proteins other than LRIG3, with a score of 276.97. The top 10 proteins were LRIG3, NETO2, OAS2, GRPEL1, DNAJA3, HAX1, MCM5, OAS1A, ELOB, NFS1.

The IHC analysis of human GBM tissues showed that NETO2 expression was moderately correlated with LRIG3 and negatively correlated with M2-like marker CD163 (Fig. 4b, c). GBM patients with high NETO2 expression had better survival than those owning low NETO2 expression (Fig. 4d). Immunofluorescence results showed that NETO2 and LRIG3 were co-located in BMDM-derived TAMs (Fig. 4e). In accordance with the results of mass spectrometry and immunofluorescence, endogenous and exogenous IP assays validated the interaction between sLRIG3 and NETO2 (Fig. 4f, g). The above results indicated that sLRIG3 interacts with NETO2 of TAMs, which may affect glioma growth by mediating the function of TAMs through NETO2.

### Soluble LRIG3 mediates NETO2 to inhibit the M2-like polarity transformation of TAMs in GBM

**Fig. 5:**
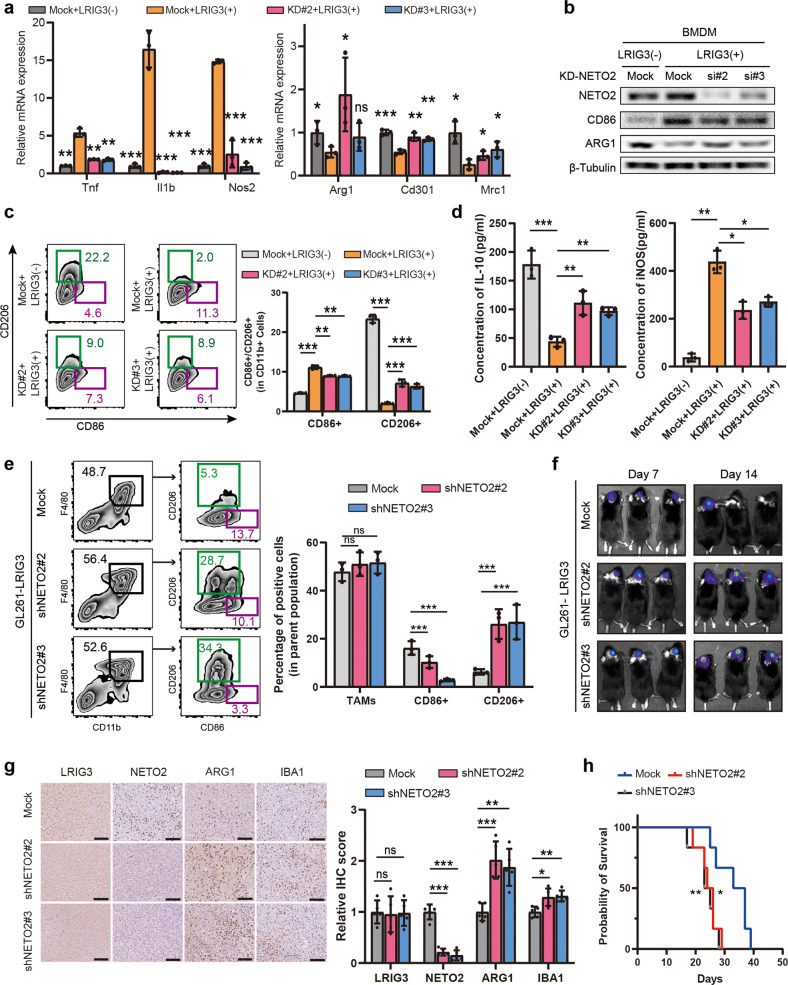
Soluble LRIG3 mediates NETO2 to inhibit the M2-like polarity transformation of TAMs in GBM. **a** qRT-PCR results of genes encoding TAM M1/M2 marker proteins in BMDM-derived TAMs transduced with Mock, siNETO2#2 and siNETO2#3 plasmids and treated with sLRIG3(−) or sLRIG3(+) CM. Values are expressed as relative expression levels to housekeeping gene GAPDH. *n* = 3 biological replicates. One-way ANOVA with bonferroni correction. **b** Immunoblots for the expression of NETO2 and TAM M1 marker CD86 and M2 marker ARG1 in BMDM-derived TAMs transduced with Mock, siNETO2#2 and siNETO2#3 plasmids and treated with sLRIG3(−) or sLRIG3(+) CM. **c** Left panel, representative images of flow cytometry for CD86+ TAMs and CD206+ TAMs in BMDM-derived TAMs transduced with Mock, siNETO2#2 and siNETO2#3 plasmids and treated with sLRIG3(−) or sLRIG3(+) CM. Right panel, quantification of CD86+ TAMs and CD206+ TAMs in BMDM-derived TAMs transduced with Mock, siNETO2#2 and siNETO2#3 plasmids and treated with sLRIG3(−) or sLRIG3(+) CM. *n* = 3 biological replicates. One-way ANOVA with bonferroni correction. **d** ELISA for the expression of IL-10 (left panel) and iNOS (right panel) protein in the supernatant medium of BMDM-derived TAMs transduced with Mock, siNETO2#2 and siNETO2#3 plasmids and treated with sLRIG3(−) or sLRIG3(+) CM. *n* = 3 biological replicates. One-way ANOVA with bonferroni correction. **e** Left panel, representative images of flow cytometry for the percentage of TAMs (CD45 + CD11b + F4/80+) in leukocytes (CD45+) and the percentage of CD86+ or CD206+ TAMs in TAMs in tumor-bearing C57BL/6 mice of different group (GL261 transduced with LRIG3 plasmid and TAMs transduced with Mock, siNETO2#2 or siNETO2#3 plasmids); Right panel, quantification of TAMs in leukocytes and CD86+ and CD206+ TAMs in TAMs in tumor-bearing C57BL/6 mice. *n* = 3 biological replicates. One-way ANOVA with bonferroni correction. **f** Representative luciferase images of three mice per group 7 and 14 days after implantation. GL261-luciferase (1.5 × 10^5^ cells) cells were transduced with LRIG3 plasmid. TAMs (5 × 10^4^ cells) were transduced with Mock, siNETO2#2, or siNETO2#3 plasmids. Color scale for GL261 cells: Min = 2.0e5; Max = 5.0e7. **g** Left panel, immunohistochemistry (IHC) for LRIG3, NETO2, ARG1, and IBA1 in GBM established from GL261 transduced with LRIG3 plasmid and TAMs transduced with Mock, siNETO2#2 or siNETO2#3 plasmids in C57BL/6 mice. Scale bar, 50 mm. Right panel, quantification of Immunohistochemistry (IHC) score for LRIG3, ARG1, and IBA1 in tumor-bearing C57BL/6 mice. (*n* = 5/group). One-way ANOVA with bonferroni correction. **h** Kaplan–Meier survival curves of C57BL/6 mice implanted with GL261 (1.5 × 10^5^ cells) transduced with LRIG3 plasmid and TAMs (5 × 10^4^ cells) transduced with Mock, siNETO2#2 or siNETO2#3 plasmids. (*n* = 6/group). Log-rank test.

In GBM, TAMs were recruited by tumor cells and polarized toward tumor-promoting M2-like TAMs, thus promoting tumor growth and progression. To investigate whether NETO2 can inhibit GBM tumor growth by inhibiting the M2 polarization of TAMs, we knocked down NETO2 in TAMs derived from BMDM and RAW264.7 cell line (Supplementary Fig. 3a). qPCR results showed that, compared with control group, silencing NETO2 expression uprugulated M2-like TAM marker signatures including *Arg1*, *Cd301* and *Mrc1*, while M1-like TAM marker signatures including *Tnf*, *Il1b* and *Nos2*(Fig. 5a and Supplementary Fig. 3b). Similarly, Western bloting (Fig. 5b and Supplementary Fig. 3c), ELISA (Fig. 5d and Supplementary Fig. 3d) and flow cytometry assays of cell lines (Fig. 5c and Supplementary Fig. 3e, f) and tumor single cell suspension (Fig. 5e) proved that, compared with the control group, the low expression of NETO2 in TAMs could block the effect of sLRIG3 and help maintain M2-like polarity transformation of TAMs, while the signature of M1-like markers decreased.

Since RAW264.7 cell line possess the main characteristics of macrophage and has been widely used as a macrophage model in many macrophage models, we used RAW264.7 to mimic TAMs in glioma microenvironment for our in vivo study. Co-implantation of TAMs with glioma tumor cells can effectively promote the growth of tumor in vivo [27], demonstrated that macrophage-like cells mimic the effect of M2-like TAMs in GBM. TAMs expressing shNETO2 or shMock and GL261 expressing LRIG3 and luciferase were co-implanted into the brain of C57 mice. Notably, bioluminescent imaging showed that silencing NETO2 promoted glioma growth (Fig. 5f), and immunohistochemical analysis confirmed that NETO2 was significantly downregulated in glioma allografts co-implanting GL261-LRIG3 and TAM-shNETO2 (Fig. 5g). Compared with the control group, the expression of M2-like marker ARG1 and total TAM marker IBA1 were both upregulated in glioma allografts co-implanted with GL261-LRIG3 and TAM-shNETO2 (Fig. 5g). Notably, the signature of CD8+ T cell marker CD8A decreased in shNETO2#2 and shNETO2#3 groups compared with control group (Supplementary Fig. 5c, d). Consistenly, co-implantation of TAM-shNETO2 with GL261-LRIG3 promoted tumor growth in vivo and was correlated with a poor prognosis (Fig. 5h). However, transwell migration assays showed that NETO2 inhibition in TAMs did not impair the capability of TAM migration (Supplementary Fig. 3g–i). Taken together, these results demonstrated that sLRIG3-NETO2 signalling is vital for suppressing the M2-like polarity transformation of TAMs and GBM progression.

### The CUB1 domain plays a vital role in the interaction of sLRIG3-NETO2 and polarity transformation of TAMs

**Fig. 6:**
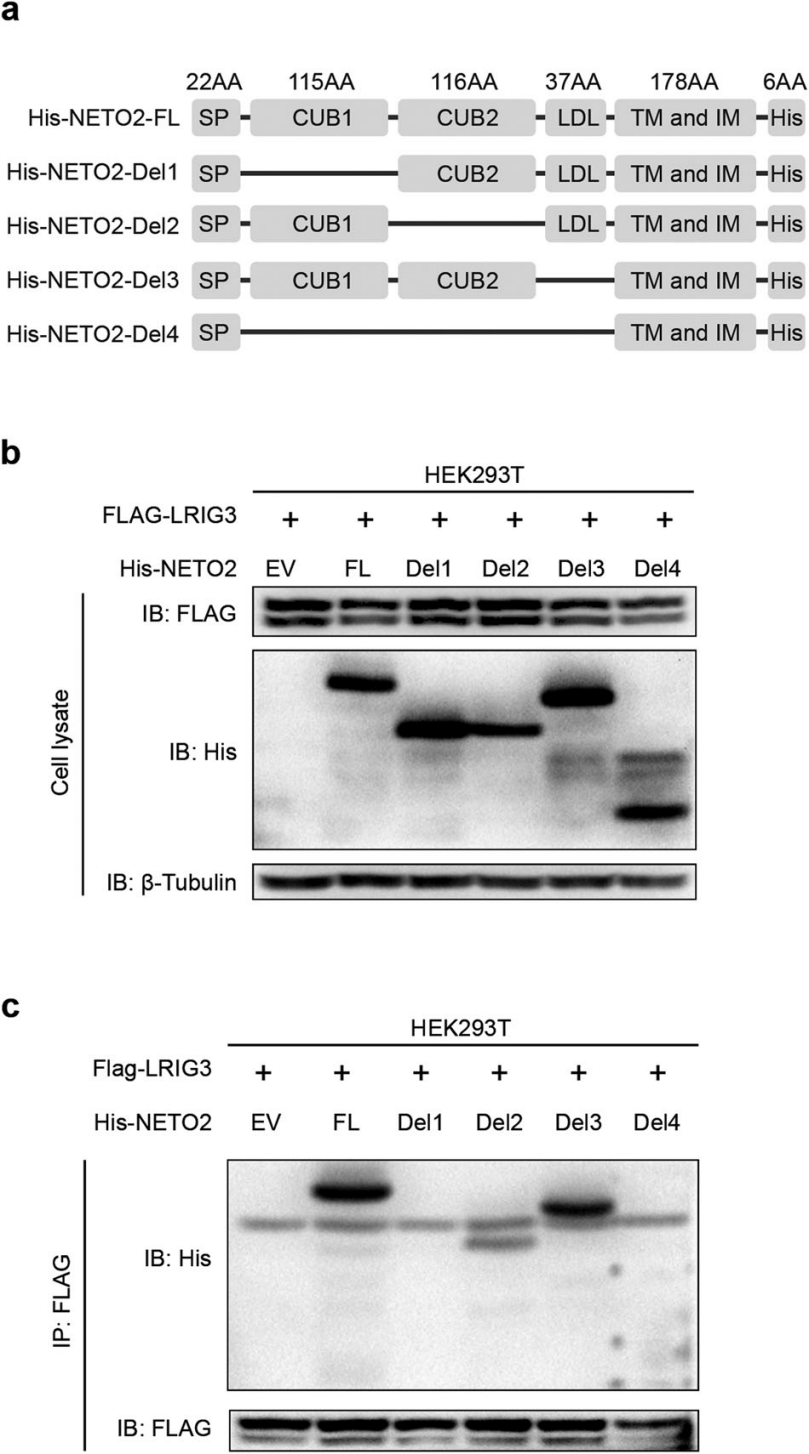
sLRIG3 interacts with the CUB1 domain of NETO2 in vitro. **a** Diagram of full-length Neto2 plasmid and four deletion mutant Neto2 plasmids including His-NETO2-FL, His-NETO2-Del1 (deletion of CUB1 domain), His-NETO2-Del2 (deletion of CUB2 domain), His-NETO2-Del3 (deletion of LDL domain), and His-NETO2-Del4 (deletion of the extracellular segment). AA: amino acid; SP: signal peptide; TM and IM: transmembrane and intramembrane. **b** Immunoblots for the expression of FLAG-LRIG3 and His-NETO2 in the HEK293T transduced with pLVX-FLAG-LRIG3 and empty vector (EV) or full-length Neto2 plasmid (FL) or four pcDNA3.1-His-NETO2 plasmids (Del1, Del2, Del3, Del4) in Fig. 6a. β-Tubulin was used as a loading control. **c** Co-immunoprecipitation of five His-NETO2 with the FLAG-specific antibody from cell lysates of HEK293T co-transduced with pLVX-FLAG-LRIG3 and empty vector (EV) or five pcDNA3.1-His-NETO2 plasmids.

To clarify the specific sites of interaction between sLRIG3 and NETO2, we constructed a full-length *Neto2* plasmid and four deletion mutant *Neto2* plasmids including His-NETO2-FL, His-NETO2-Del1 (deletion of CUB1 domain), His-NETO2-Del2 (deletion of CUB2 domain), His-NETO2-Del3 (deletion of LDL domain), and His-NETO2-Del4 (deletion of the extracellular segment) (Fig. 6a). We co-transfected FLAG-LRIG3 and five full-length or truncated mutant plasmids of *Neto2* in 293 T cells. Exogenous IP assay demonstrated that the protein of His-NETO2 lacking the CUB1 domain or the extracellular segment of NETO2 lost the ability to interact with sLRIG3 (Fig. 6b, c). These results suggest that sLRIG3 binds to the CUB1 domain of NETO2. Interestingly, it seems that the deletion of the CUB2 domain might partly block the binding of NETO2 and sLRIG3.

**Fig. 7:**
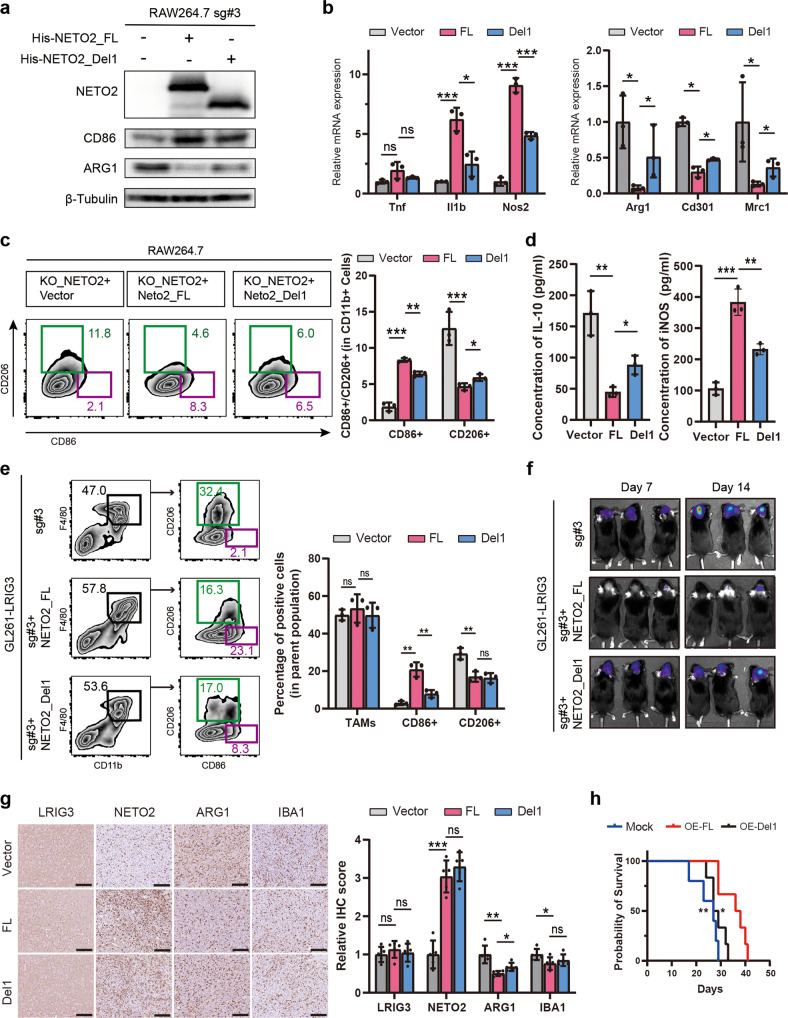
The CUB1 domain plays a vital role in the interaction of sLRIG3-NETO2 and polarity transformation of TAMs. **a** Immunoblots for the expression of NETO2, TAM M1 marker CD86 and M2 marker ARG1 in RAW264.7-derived TAMs co-transduced sg-NETO2#3 with Mock, His-NETO2-FL or His-NETO2-Del1 plasmids. **b** qRT-PCR results of genes encoding TAM M1/M2 marker proteins in RAW264.7-derived TAMs co-transduced sg-NETO2#3 with Mock, His-NETO2-FL or His-NETO2-Del1 plasmids. Values are expressed as relative expression levels to housekeeping gene GAPDH. *n* = 3 biological replicates. One-way ANOVA with bonferroni correction. **c** Left panel, representative images of flow cytometry for CD86+ TAMs and CD206+ TAMs in RAW264.7-derived TAMs co-transduced sg-NETO2#3 with Mock, His-NETO2-FL or His-NETO2-Del1 plasmids. Right panel, quantification of CD206+ TAMs in RAW264.7-derived TAMs co-transduced sg-NETO2#3 with Mock, His-NETO2-FL or His-NETO2-Del1 plasmids. *n* = 3 biological replicates. One-way ANOVA with bonferroni correction. **d** ELISA for the expression of IL-10 (left panel) and iNOS (right panel) protein in the supernatant medium of RAW264.7-derived TAMs co-transduced sg-NETO2#3 with Mock, His-NETO2-FL or His-NETO2-Del1 plasmids. *n* = 3 biological replicates. One-way ANOVA with bonferroni correction. **e** Left panel, representative images of flow cytometry for the percentage of TAMs (CD45 + CD11b + F4/80+) in leukocytes (CD45+) and the percentage of CD86+ or CD206+ TAMs in TAMs in tumor-bearing C57BL/6 mice of different group (GL261 transduced with LRIG3 plasmid and TAMs co-transduced sg-NETO2#3 with Mock, His-NETO2-FL or His-NETO2-Del1 plasmids); Right panel, quantification of TAMs in leukocytes and CD86+ and CD206+ TAMs in TAMs in tumor-bearing C57BL/6 mice. *n* = 3 biological replicates. One-way ANOVA with bonferroni correction. **f** Representative luciferase images of three C57BL/6 mice per group 7 and 14 days after implantation. GL261-luciferase (1.5 × 10^5^ cells) cells were transduced with LRIG3 plasmid. TAMs (5 × 10^4^ cells) were co-transduced sg-NETO2#3 with Mock, His-NETO2-FL, or His-NETO2-Del1 plasmids. Color scale for GL261 cells: Min = 2.0e5; Max = 5.0e7. **g** Immunohistochemistry (IHC) for LRIG3, NETO2, ARG1, and IBA1 in GBM established from GL261 transduced with LRIG3 plasmid and TAMs co-transduced sg-NETO2#3 with Mock, His-NETO2-FL or His-NETO2-Del1 plasmids in C57BL/6 mice. Scale bar, 100 mm. (*n* = 5/group). One-way ANOVA with bonferroni correction. **h** Kaplan–Meier survival curves of C57BL/6 mice implanted with GL261 (1.5 × 10^5^ cells) transduced with LRIG3 plasmid and TAMs (5 × 10^4^ cells) co-transduced sg-NETO2#3 with Mock, His-NETO2-FL or His-NETO2-Del1 plasmids. (Mock (*n* = 5/group), His-NETO2-FL (*n* = 6/group), His-NETO2-Del1 (*n* = 6/group)). Log-rank test.

To further explore whether the CUB1 domain of NETO2 can exert its inhibitory effect on tumor growth by changing the M2-like polarity transformation of TAMs, endogenic NETO2 was knocked out via CRISPR in RAW264.7-derived TAMs and His-NETO2-FL or His-NETO2-Del1 was transfected into targeted cells (Fig. 7a and Supplementary Fig. 4a). The results of qPCR (Fig. 7b) showed that the expression of M2-like TAM markers, (*Arg1*, *Cd301,* and *Mrc1*) decreased, while the expression of M1-like TAM markers (*Tnf*, *Il1b,* and *Nos2*) increased in TAMs transfected with His-NETO2-Del1, compared with those transfected with full-length *Neto2* plasmid (Fig. 7c). Similarly, Western bloting (Fig. 7c), ELISA (Fig. 7d) and flow cytometry assays of cell lines (Fig. 7c) and tumor single cell suspension (Fig. 7e) demonstrated that exogenous expression of full-length *Neto2* plasmids recovered the capability of sLRIG3 to attenuate the tilt towards an M2-dominant TAM population compared with TAMs transfected of empty or CUB1-deleted *Neto2* plasmids. Interestingly, TAMs transfected with His-NETO2-Del1 plasmid seem to partially recover the ability of sLRIG3 in TAM polarity transformation (Fig. 7b–e), suggesting that CUB1-deleted NETO2 might inhibit the M2-like polarity transformation of TAMs through other mechanisms.

GL261 expressing LRIG3 and luciferase and TAMs expressing Mock, His-NETO2-FL or His-NETO2-Del1 were co-implanted into the brain of C57 mice. Bioluminescence imaging showed that compared with blank or His-NETO2-Del1 group, TAMs with His-NETO2-FL recovered the effects of sLRIG3 inhibiting glioma growth (Fig. 7f). The survival data of each group was consistent with the results of tumor growth (Fig. 7h). Consistently, immunohistochemical analysis confirmed that M2-like marker ARG1 and total TAM marker IBA1 were downregulated, while the CD8+ T cells marker CD8A increased in glioma allografts co-implanted with GL261-sLRIG3 and TAMs with exogenous full-length NETO2, compared with the blank or His-NETO2-Del1 group (Fig. 7g and Supplementary Fig. 5e, f). These results demonstrated that the CUB1 domain of NETO2 plays an important role in sLRIG3’s reversal effect of M2 TAM polarization, thus remodelling the heterogeneous GBM microenvironment and inhibiting tumor growth.

### sLRIG3–NETO2 signalling activates the NF-kB pathway in TAMs of GBM

To explore downstream pathways mediating sLRIG3-NETO2 signalling in TAMs, we performed RNA-sequencing analysis on TAMs treated with sLRIG3(+) or sLRIG3(−) supernatant. KEGG enrichment analysis based on the RNA-sequencing data of BMDM-derived TAMs treated with sLRIG3(+) or sLRIG3(−) supernatant showed that PD-1 and PD-L1 immune checkpoint pathway, NF-kB signaling pathway, apoptosis-related pathway, Toll-like receptor pathway and insulin signaling pathway were enriched in sLRIG3(+) TAMs (Supplementary Fig. 4b). In accordance with the silico results, western blotting showed that, sLRIG3(+) medium increased p-P65 and promote nuclear translocation of P65 in TAMs (Supplementary Fig. 4c). In addition, Jsh-23, a specific inhibitor of NF-kB pathway, can block the activation of NF-kB pathway by sLRIG3 in TAMs of GBM (Supplementary Fig. 4d).

In NETO2-knockout TAMs, exogenous expression of full-length NETO2 upregulated the expression of p-P65 and nuclear P65, compared with TAMs transfected with empty or CUB1-deletion plasmids (Supplementary Fig. 4e). Interestingly, partial NF-kB pathway activation was also observed in TAMs with exogenous His-NETO2-Del1, compared with the empty group (Supplementary Fig. 4e).

Considering that the apoptosis-related pathway was enriched in KEGG enrichment analysis, FITC AnnexinV (BD Pharmingen) and Fixable Viability Stain 780 (BD Pharmingen) were used to assess apoptosis of RAW264.7-derived TAMs with stimulation of sLRIG3(+) or sLRIG3(−) CM (Supplementary Fig. 6a). Although there is statistically significant difference between two groups in late apoptosis, the difference is too small to account for the reduced number of IBA1+ macrophages. Further, the protein expression of BCL2 and BAX in brain tumors of GL261-Vector-bearing or GL261-LRIG3-bearing mice were detected (Supplementary Fig. 6b). Interestingly, the results showed that the scores of BCL2/BAX was higher in the LRIG3 group compared to the Vector group (but with no statistical difference). All these apoptosis-related data support that there were no biological differences of the number of apoptotic macrophages between the sLRIG3(+) and sLRIG3(−) groups.

In conclusion, the evidence above proved that sLRIG3-NETO2 signalling mediated M2-like polarity transformation of TAMs in GBM through the NF-kB pathway, thus affecting the growth and prognosis of gliomas.

## Discussion

In GBM microenvironment, various heterogeneous cells, containing tumor cells and TAMs, interact with each other to regulate the malignant progression of tumors [28, 29]. Our previous study has discovered sLRIG3 in glioma cystic fluid and serum of glioma patients, and sLRIG3 in serum of glioma patients was positively correlated with the grade of glioma [17]. The expression level of serum sLRIG3 was positively correlated with prognosis in high-grade gliomas. The current evidence suggests that sLRIG3 may play a significant role in the GBM TME and supress glioma progression. In this study, we found that GBM tumor cells released LRIG3 to a soluble form through the regulation of ADAM17 and attenuate the tilt towards an M2-dominant TAM population, thus remodelling the tumor microenvironment and suppressing tumor growth. Knocking down NETO2 by shRNA or knocking out NETO2 by CRISPR can block the interaction of sLRIG3 and NETO2, thus largely abrogating the tumor-suppressive effects of sLRIG3. It is crucial to further identify the detailed targets of interaction between sLRIG3 and NETO2 and how they exert their tumor-suppressive effects in GBM TME. Our further studies showed that the CUB1 domain of NETO2 plays an important role in sLRIG3-NETO2 interaction and regulates the M1/M2 polarity transformation of TAMs. Moreover, overexpressing His-target NETO2 with CUB1 deletion mutation does not fully recover the suppressive effects of sLRIG3 on the M1/M2-polarization in NETO2-Knockout TAM model. This study suggests that sLRIG3, modulated by ADAM17 in GBM tumor cells, interacts with the CUB1 domain of NETO2 and plays a key role in attenuating the tilt towards an M2-dominant TAM population, thus remodelling the heterogeneous glioblastoma microenvironment, and suppressing GBM malignant growth.

GBM contains abundant tumor-supporting TAMs to promote tumor malignant progression, and the reciprocal interaction between tumor cells and TAMs is one of the crucial factors for GBM therapeutic resistance and tumor recurrence [30, 31]. GBM tumor cells recruit TAMs and regulate the biology of TAMs through secreting a variety of soluble proteins [32, 33]. On the other hand, heterogeneous TAMs play a vital role in regulating the proliferation, invasion, and angiogenesis of tumor cells, thus orchestrating an intricate TME and regulating GBM progression [27, 34, 35]. Our study verified that sLRIG3 derived from tumor cells can suppress the malignant progression of GBM through restraining the recruitment of TAMs and attenuating the tilt towards an M2-dominant TAM population, demonstrating that sLRIG3 is a vital molecular mediator for the interaction between GBM tumor cells and TAMs in the GBM TME. Moreover, soluble proteins derived from GBM tumor cells can affect TAMs to achieve the capability of immune escape [36–38]. Studies have shown that TAMs in TME can inhibit the functions of Th1 and cytotoxic T cell and recruit Tregs and MDSCs through secreting CCL2, TGF-β and IL-10, thus orchestrating an immunosuppressive TME. Further studies of the interaction between tumor cells and TAMs in the TME will help us understand the crucial role of TAMs in regulating the immunosuppressive microenvironment of GBM and may help improve our understanding of GBM immunotherapy.

Tumor immunotherapy, containing (ICB) agents, therapeutic vaccines, adoptive cell therapy, monoclonal antibody (mAbs) and oncolytic virus, is a promising strategy for tumor therapy [39, 40]. Currently some immunotherapy strategies have been used to treat cancer and made remarkable achievements in recurrent Squamous-Cell Carcinoma, melanomas, and other tumors [41–43]. However, immunotherapy remains a significant challenge for GBM, due to the immunosuppressive microenvironment, which is at least partially mediated by TAMs [44, 45]. Therefore, targeting TAMs may improve the efficacy of GBM immunotherapy. Currently, the existing GBM treatment strategies targeting TAMs include inhibiting the recruitment of TAMs [11], attenuating the polarization of tumor-promoting TAMs [16] and eliminating tumor-promoting TAMs [38, 46]. Our study demonstrated that sLRIG3 could reverse M2-like polarization of TAMs through the interaction with NETO2, suggesting that sLRIG3 and NETO2 may be TAM-related therapeutic targets. Moreover, the extracellular CUB1 domain of NETO2 plays a crucial role in the interaction with sLRIG3 in inhibiting the polarization of TAMs toward M2. The development of small-molecule drugs targeting the key domain may provide new strategies for targeting TAMs. It is important to note that current phase III clinical trials using single immunotherapy approaches, such as ICB [47] and therapeutic vaccines [48, 49], failed to improve the OS of GBM patients. In contrast, immunotherapy combined with modified chemotherapy or other multiple immunotherapies have shown promising clinical activities and achieved encouraging success [50–52], which suggests that immunotherapy scheme for GBM may require the combination of multistep process to improve the therapeutic effect. Targeting multiple arms of GBM may be a promising strategy. Therefore, the combination of small molecule drugs targeting potential TAMs targets sLRIG3 and NETO2 with current immunotherapy such as ICBs, therapeutic vaccines, and traditional chemotherapy may be a promising strategy to improve the overall survival of GBM patients.

Our previous studies have demonstrated that LRIG3 and sLRIG3 downregulate the MET/ Phosphatidylinositol 3-kinase/Akt signaling pathway in glioma tumor cells and inhibit GBM proliferation and invasion [17]. In GBM TME, soluble proteins secreted by glioma tumor cells can affect the biology including polarity transformation [53, 54], migration [55, 56], proliferation, and apoptosis [30, 57] of TAMs, thus regulating the progression of GBM. In this study, we performed GSEA analysis based on the TCGA GBM datasets and demonstrated that high LRIG3 expression level was correlated with active immune-related pathways, high gene set signatures of CD8+ T cells, and low gene set signatures of M1/M2 macrophages. In addition, sLRIG3 derived from GBM tumor cells can suppress the migration of TAMs and attenuate the tilt toward an M2-dominant TAM population. Interestingly, knocking down/out NETO2 blocked the sLRIG3’s effect of attenuating the M2-like polarity transformation, while the migration ability of TAMs did not decrease. Although the apoptosis-related pathway was enriched in the results of our RNA sequencing, experiments data showed that apoptosis may not explain the reduced number of TAMs in sLRIG3(+) group, while the downregulation of interhrin beta3 in sLRIG3(+) group TAMs offered a potential explanation of this phenomenon. Future research in subsequent studies would be performed to investigate the effect of LRIG3 on cell migration and its mechanism. Moreover, knocking out NETO2 by CRISPR can not fully abrogate sLRIG3’s effect towards attenuating the M2-like polarity transformation of TAMs. The evidence above suggests that sLRIG3 may affect TAMs through multiple arms including interacting with NETO2. sLRIG3 in the TME may perform crucial effects through multiple stromal or immune cells to regulate the GBM progression. It is worth noting that knocking out NETO2 of TAM in this study promotes the GBM progression through at least partly blocking the tumor-suppressive effect of sLRIG3. In addition, NETO2 expression was upregulated in multiple cancer and was correlated with the prognosis of cancer patients [20–22], which suggests that NETO2 may affect the progression of different cancer through different mechanisms. sLRIG3 may play an intricate role in GBM TME, while NETO2 might cause an opposite tumor biological effect through different mechanisms in various malignant tumors. Deep mechanisms need to be further studied. In conclusion, our study found that both sLRIG3 and NETO2 may affect tumor progression through multiple pathways. sLRIG3, as a potential therapeutic target, may affect the biology of TAMs in TME through multiple arms, and suppress the malignant progression of GBM. Therapy targeting sLRIG3 may affect the growth of GBM from multiple dimensions and achieve better efficacy.

## Supplementary information

Figure legends of supplementary figures

Supplementary figure 1

Supplementary figure 2

Supplementary figure 3

Supplementary figure 4

Supplementary figure 5

Supplementary figure 6

Supplementary table 1

Supplementary table 2

Supplementary table 3

Supplementary table 4

Supplementary table 5

Author Contribution Statement

Reproducibility checklist

Original Data File of WB bands in figure 2

Original Data File of WB bands in figure 2-2

Original Data File of WB bands in figure 3

Original Data File of WB bands in figure 4

Original Data File of WB bands in figure 5

Original Data File of WB bands in figure 6

Original Data File of WB bands in figure 7

Original Data File of WB bands in supplementary figure 1

Original Data File of WB bands in supplementary figure 1–2

Original Data File of WB bands in supplementary figure 2

Original Data File of WB bands in supplementary figure 2–2

Original Data File of WB bands in supplementary figure 3a

Original Data File of WB bands in supplementary figure c

Original Data File of WB bands in supplementary figure 4

Original Data File of WB bands in supplementary figure 4–2

Original Data File of WB bands in supplementary figure 4–3

Original Data File of WB bands in supplementary figure 4–4

Original Data File of WB bands in supplementary figure 4–5

## Data Availability

Data of RNA sequence have been submitted to NCBI SRA database. The accession number is PRJNA833258. The raw data of this study are available from the corresponding author upon reasonable request.
