# Supplementary material for: Glioma-derived LRIG3 interacts with NETO2 in tumor-associated macrophages to modulate microenvironment and suppress tumor growth
Source: Cell Death Dis. 2023 Jan 13;14(1):28. doi: 10.1038/s41419-023-05555-z (PMC9839712; doi:10.1038/s41419-023-05555-z)
Supplement: Supplementary file 1 — Figure legends of supplementary figures [file 41419_2023_5555_MOESM1_ESM.docx]

**Supplementary figure legend**

**Figure S1. Immunoblots of soluble LRIG3 in LRIG3-overexpressing (OE) GL261 cells treated with siADAM17, ADAM17** **agonist and ADAM17 inhibitor.**

(a) Immunoblots for LRIG3 in the cell lysates of control and LRIG3-overexpressing (OE) GL261 cells.

(b) Immunoblots for the expression of soluble LRIG3 protein of supernatant medium in LRIG3-overexpressing (OE) GL261 cells with stimulation of ADAM17 agonist PMA, ADAM17 inhibitor TAPI and ADAM17 siRNAs.

(c) Immunoblots for the expression of soluble LRIG3 protein of supernatant medium in LRIG3-overexpressing (OE) GL261 cells in the absence or presence of increasing doses of ADAM17 agonist PMA.

(d) Immunoblots for the expression of soluble LRIG3 protein of supernatant medium in LRIG3-overexpressing (OE) GL261 cells in the absence or presence of increasing doses of ADAM17 inhibitor TAPI.

**Figure S2. Soluble LRIG3 inhibits the M2 polarization of TAMs to suppress GBM progression.**

(a) qRT-PCR for genes encoding TAM M1/M2 marker proteins in RAW264.7-derived TAMs treated with sLRIG3(+) or sLRIG3(-) CM. Values are expressed as relative expression levels to housekeeping gene GAPDH. n = 3 biological replicates. Unpaired student's t test.

(b) Immunoblots for the expression of TAM M1 marker CD86 and M2 marker ARG1 in RAW264.7-derived TAMs treated with sLRIG3(+) or sLRIG3(-) CM.

(c) Left panel, representative images of flow cytometry for CD86+ TAMs and CD206+ TAMs in RAW264.7-derived TAMs treated with sLRIG3(+) or sLRIG3(-) CM. Right panel, quantification of CD86+ TAMs and CD206+ TAMs in RAW264.7-derived TAMs treated with sLRIG3(+) or sLRIG3(-) CM. n = 3 biological replicates. Unpaired student's t test.

(d) Elisa for the expression of IL-10 (left panel) and iNOS (right panel) protein in the supernatant medium of RAW264.7-derived TAMs treated with sLRIG3(+) or sLRIG3(-) CM. n = 3 biological replicates. Unpaired student's t test.

(e) Left panel, representative transwell analysis of BMDM-derived TAMs following stimulation with sLRIG3(+) or sLRIG3(-) CM. Right panel, quantification of relative migration of BMDM-derived TAMs following stimulation with sLRIG3(+) or sLRIG3(-) CM. Scale bar, 300 μm. n = 3 biological replicates. Unpaired student's t test.

(f) Left panel, representative transwell analysis of RAW264.7-derived TAMs following stimulation with sLRIG3(+) or sLRIG3(-) CM. Right panel, quantification of relative migration of RAW264.7-derived TAMs following stimulation with sLRIG3(+) or sLRIG3(-) CM. Scale bar, 300 μm. n = 3 biological replicates. Unpaired student's t test.

(g) Immunoblots for the expression of CCR2, CCR4 and ITGB3 in BMDM-derived (left panel) and RAW264.7-derived (right panel) TAMs treated with sLRIG3(+) or sLRIG3(-) CM.

**Figure S3. Soluble LRIG3 mediates NETO2 to inhibit the M2-like polarity transformation of TAMs in GBM.**

(a) Immunoblots for the expression of NETO2 in BMDM-derived and RAW264.7-derived TAMs transduced with Mock, shNETO2#1, shNETO2#2, and shNETO2#3.

(b) qRT-PCR results of genes encoding TAM M1/M2 marker proteins in RAW264.7-derived TAMs transduced with Mock, shNETO2#2 and shNETO2#3 plasmids and treated with sLRIG3(-) or sLRIG3(+) CM. Values are expressed as relative expression levels to housekeeping gene GAPDH. n = 3 biological replicates. One-way ANOVA with bonferroni correction.

(c) Immunoblots for the expression of NETO2, TAM M1 marker CD86 and TAM M2 marker ARG1 in RAW264.7-derived TAMs transduced with Mock, shNETO2#2 and shNETO2#3 plasmids and treated with sLRIG3(-) or sLRIG3(+) CM.

(d) Elisa for the expression of IL-10 (left panel) and iNOS (right panel) protein in the supernatant medium of RAW264.7-derived TAMs transduced with Mock, shNETO2#2 and shNETO2#3 plasmids and treated with sLRIG3(-) or sLRIG3(+) CM. n = 3 biological replicates. One-way ANOVA with bonferroni correction.

(e) Representative images of flow cytometry for CD86+ and CD206+ TAMs in RAW264.7-derived TAMs transduced with Mock, shNETO2#2 and shNETO2#3 plasmids and treated with sLRIG3(-) or sLRIG3(+) CM.

(f) Quantification of CD86+ and CD206+ TAMs in RAW264.7-derived TAMs transduced with Mock, shNETO2#2 and shNETO2#3 plasmids and treated with sLRIG3(-) or sLRIG3(+) CM. n = 3 biological replicates. One-way ANOVA with bonferroni correction.

(g) Representative transwell analysis of BMDM-derived TAMs transduced with Mock, shNETO2#2 and shNETO2#3 plasmids and treated with sLRIG3(-) or sLRIG3(+) CM. Scale bar, 300 μm.

(h)Quantification of relative migration of BMDM-derived(left panel) and RAW264.7-derived(right panel) TAMs transduced with Mock, shNETO2#2 and shNETO2#3 plasmids and treated with sLRIG3(-) or sLRIG3(+) CM. n = 3 biological replicates. One-way ANOVA with bonferroni correction.

(i) Representative transwell analysis of RAW264.7-derived TAMs transduced with Mock, shNETO2#2 and shNETO2#3 plasmids and treated with sLRIG3(-) or sLRIG3(+) CM. Scale bar, 300 μm.

**Figure S4.** **sLRIG3–NETO2 signalling activates the NF-kB pathway in TAMs of GBM.**

(a) Immunoblots for the expression of NETO2 in RAW264.7-derived TAMs transduced with Mock, sg-NETO2#3 and sg-NETO2#4.

(b) Pathway enrichment analysis based on KEGG database shows the markedly enriched pathways in BMDM-derived TAMs treated with sLRIG3(+) CM compared to TAMs treated with sLRIG3(-) CM. X-axis shows adjusted p-values corrected for multiple testing.

(c) Immunoblots for the expression of p-p65, p65, β-Tubulin, Nuclear-p65 and Histone H3 in BMDM- and RAW264.7-derived TAMs treated with sLRIG3(+) or sLRIG3(-) CM.

(d) Immunoblots for the expression of p-p65, p65, β-Tubulin, Nuclear-p65 and Histone H3 in BMDM- and RAW264.7-derived TAMs transduced with blank or LRIG3 plasmids in the absence or NF-κB inhibitor JSH-23.

(e) Immunoblots for the expression of p-p65, p65, β-Tubulin, Nuclear-p65 and Histone H3 in RAW264.7-derived TAMs co-transduced sg-NETO2#3 with Mock, His-NETO2-FL or His-NETO2-Del1.

**Figure S5.** **Flow cytometry results of the percentage of CD8+ T cells in leukocytes of tumor microenvironment in tumor-bearing C57BL/6 mice of different groups.**

(a) Left panel, immunohistochemistry (IHC) for CD8A in tumor-bearing (GL261-Vector and GL261-LRIG3) C57BL/6 mice. Scale bar, 50 mm. Right panel, quantification of Immunohistochemistry (IHC) score for CD8A in tumor-bearing (GL261-Vector (n = 6/group) and GL261-LRIG3 (n = 5/group)) C57BL/6 mice. Unpaired student's t test.

(b) Left panel, representative images of flow cytometry of CD8+ T cells in leukocytes of tumor microenvironment in tumor-bearing C57BL/6 mice of different groups (GL261-Vector and GL261-LRIG3); Right panel, quantification of CD8+ T cells in leukocytes of tumor microenvironment in tumor-bearing C57BL/6 mice. n = 3 biological replicates. Unpaired student's t test.

(c) Left panel, immunohistochemistry (IHC) for CD8A in tumor-bearing C57BL/6 mice of different group (GL261 transduced with LRIG3 plasmid and TAMs transduced with Mock, siNETO2#2 and siNETO2#3 plasmids). Scale bar, 50 mm. Right panel, quantification of Immunohistochemistry (IHC) score for CD8A in tumor-bearing C57BL/6 mice. (n = 5/group). One-way ANOVA with bonferroni correction.

(d) Left panel, representative images of flow cytometry of CD8+ T cells in leukocytes of tumor microenvironment in tumor-bearing C57BL/6 mice of different groups (GL261 transduced with LRIG3 plasmid and TAMs transduced with Mock, siNETO2#2 or siNETO2#3 plasmids); Right panel, quantification of CD8+ T cells in leukocytes of tumor microenvironment in tumor-bearing C57BL/6 mice. n = 3 biological replicates. One-way ANOVA with bonferroni correction.

(e) Left panel, immunohistochemistry (IHC) for CD8A in tumor-bearing C57BL/6 mice of different group (GL261 transduced with LRIG3 plasmid and TAMs co-transduced sg-NETO2#3 with Mock, His-NETO2-FL or His-NETO2-Del1 plasmids). Scale bar, 50 mm. Right panel, quantification of Immunohistochemistry (IHC) score for CD8A in tumor-bearing C57BL/6 mice. (n = 5/group). One-way ANOVA with bonferroni correction.

(f) Left panel, representative images of flow cytometry of CD8+ T cells in leukocytes of tumor microenvironment in tumor-bearing C57BL/6 mice of different groups (GL261 transduced with LRIG3 plasmid and TAMs co-transduced sg-NETO2#3 with Mock, His-NETO2-FL or His-NETO2-Del1 plasmids); Right panel, quantification of CD8+ T cells in leukocytes of tumor microenvironment in tumor-bearing C57BL/6 mice. n = 3 biological replicates. One-way ANOVA with bonferroni correction.

**Figure S6. Soluble LRIG3 do not induce apoptosis of TAMs in tumor microenvironment.**

**(a)** Left panel, representative images of flow cytometry for the percentage of apoptotic cells in RAW264.7-derived TAMs treated with sLRIG3(+) or sLRIG3(-) CM. Right panel, quantification of the percentage of apoptotic cells in RAW264.7-derived TAMs treated with sLRIG3(+) or sLRIG3(-) CM. n = 3 biological replicates. Unpaired student's t test.

**(b)** Left panel, immunohistochemistry (IHC) for BCL2 and BAX in tumor-bearing (GL261-Vector and GL261-LRIG3) C57BL/6 mice. Scale bar, 50 mm. Right panel, quantification of Immunohistochemistry (IHC) relative score of BCL2/BAX in tumor-bearing (GL261-Vector and GL261-LRIG3) C57BL/6 mice. (n = 5/group). Unpaired student's t test.
