# Supplementary figures and images for: Glioma-derived LRIG3 interacts with NETO2 in tumor-associated macrophages to modulate microenvironment and suppress tumor growth
Source: Cell Death Dis. 2023 Jan 13;14(1):28. doi: 10.1038/s41419-023-05555-z (PMC9839712; doi:10.1038/s41419-023-05555-z)

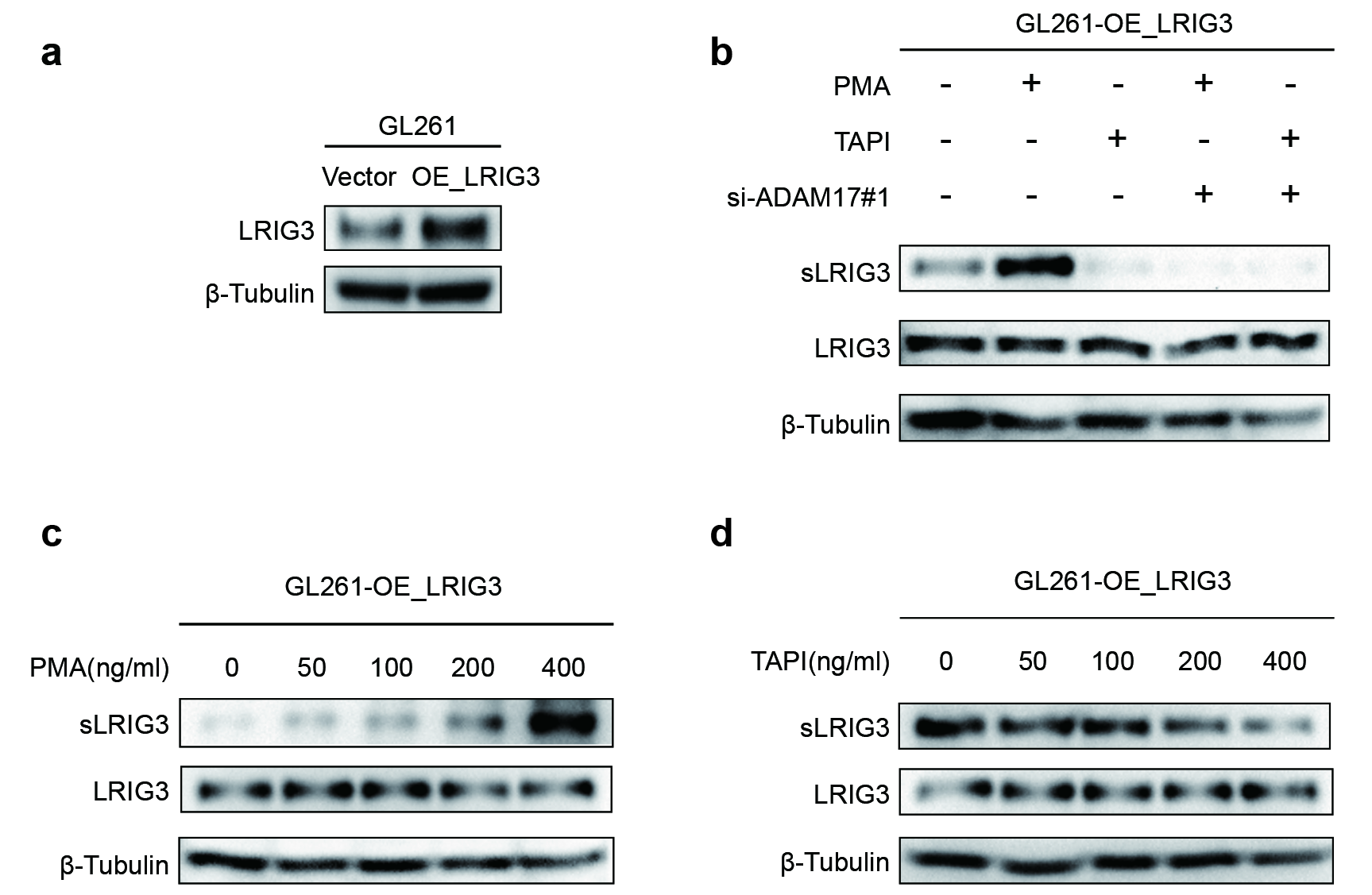

Supplement: Supplementary file 2 — Supplementary figure 1 [file 41419_2023_5555_MOESM2_ESM.tif]

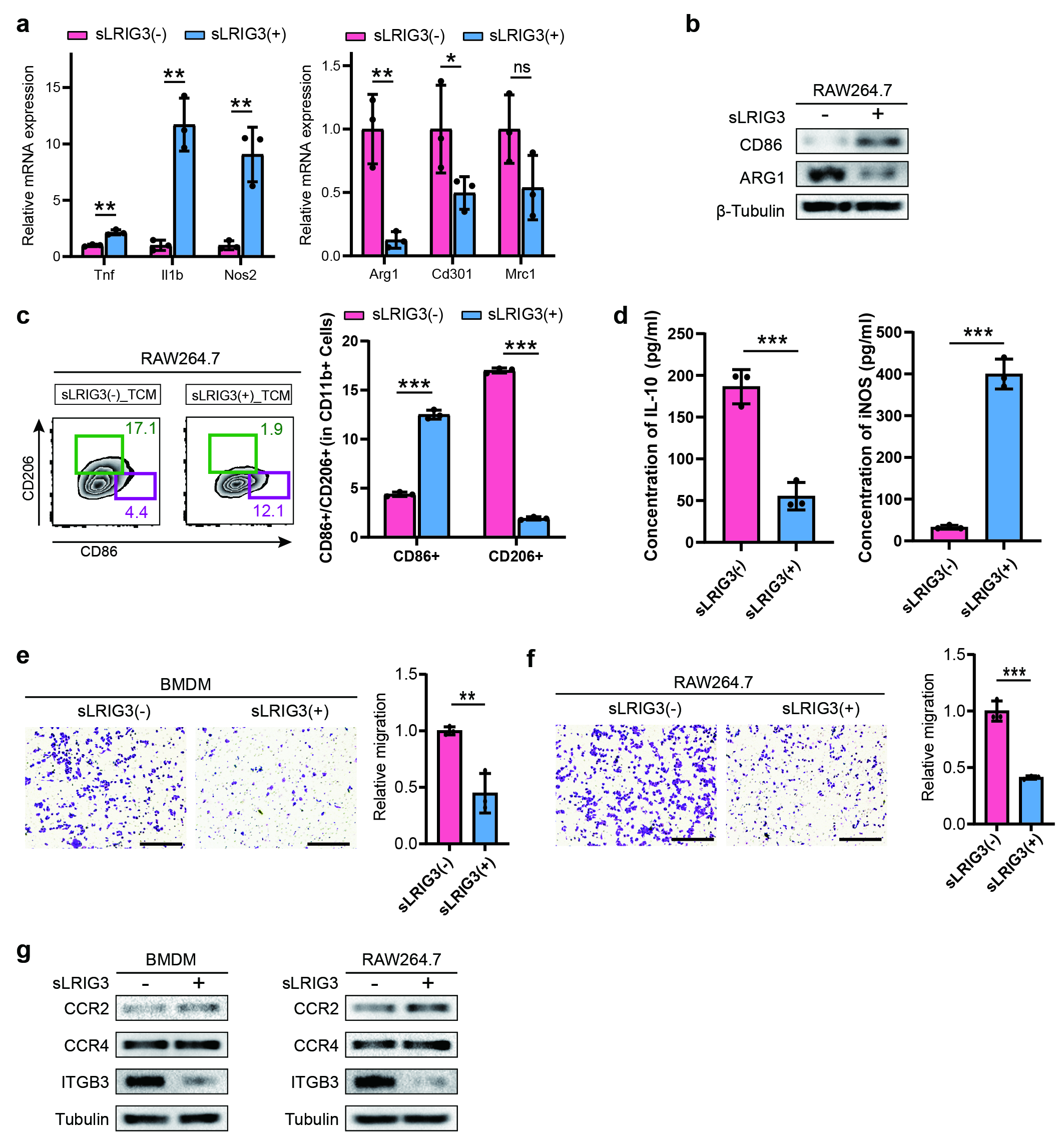

Supplement: Supplementary file 3 — Supplementary figure 2 [file 41419_2023_5555_MOESM3_ESM.tif]

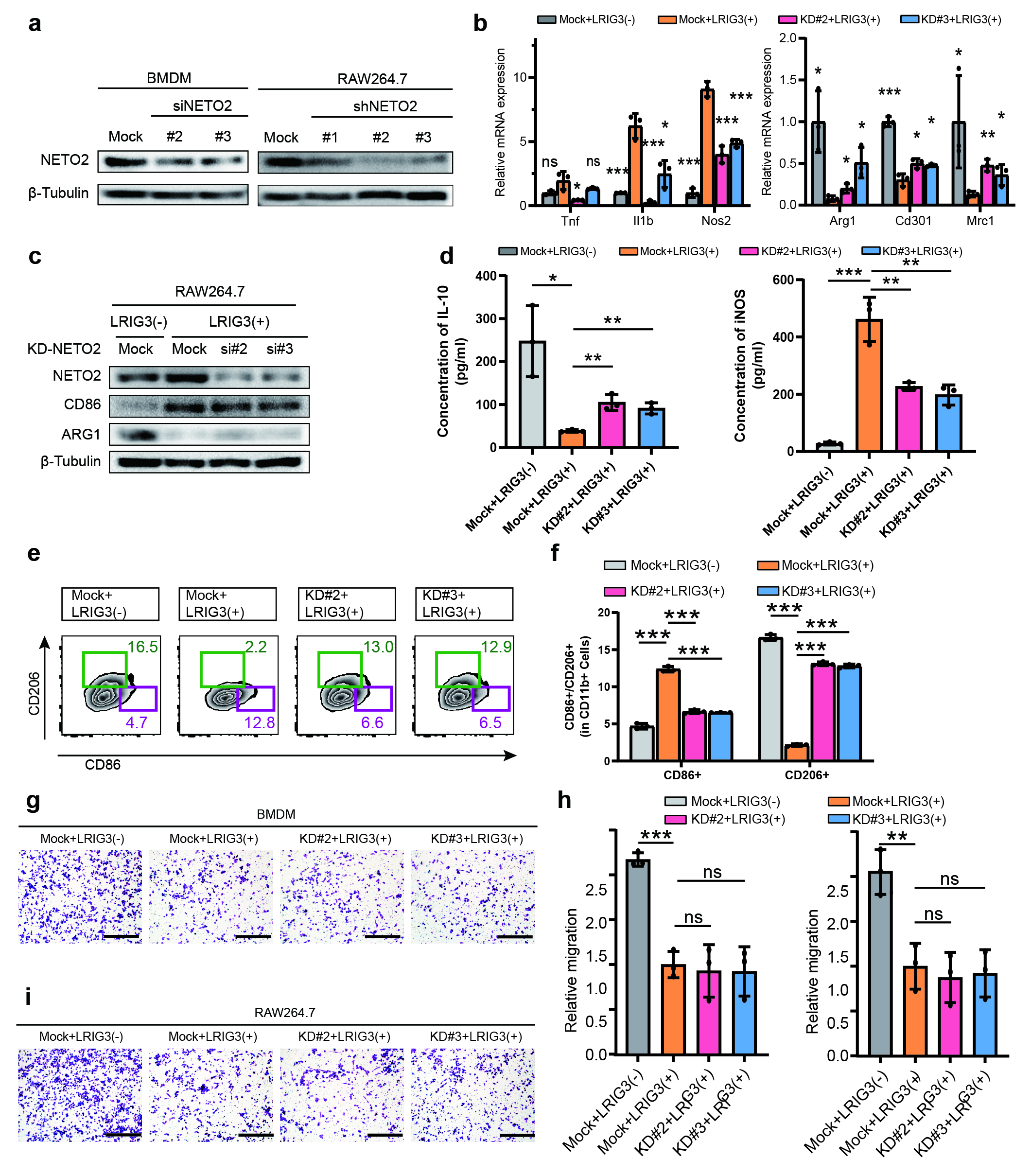

Supplement: Supplementary file 4 — Supplementary figure 3 [file 41419_2023_5555_MOESM4_ESM.tif]

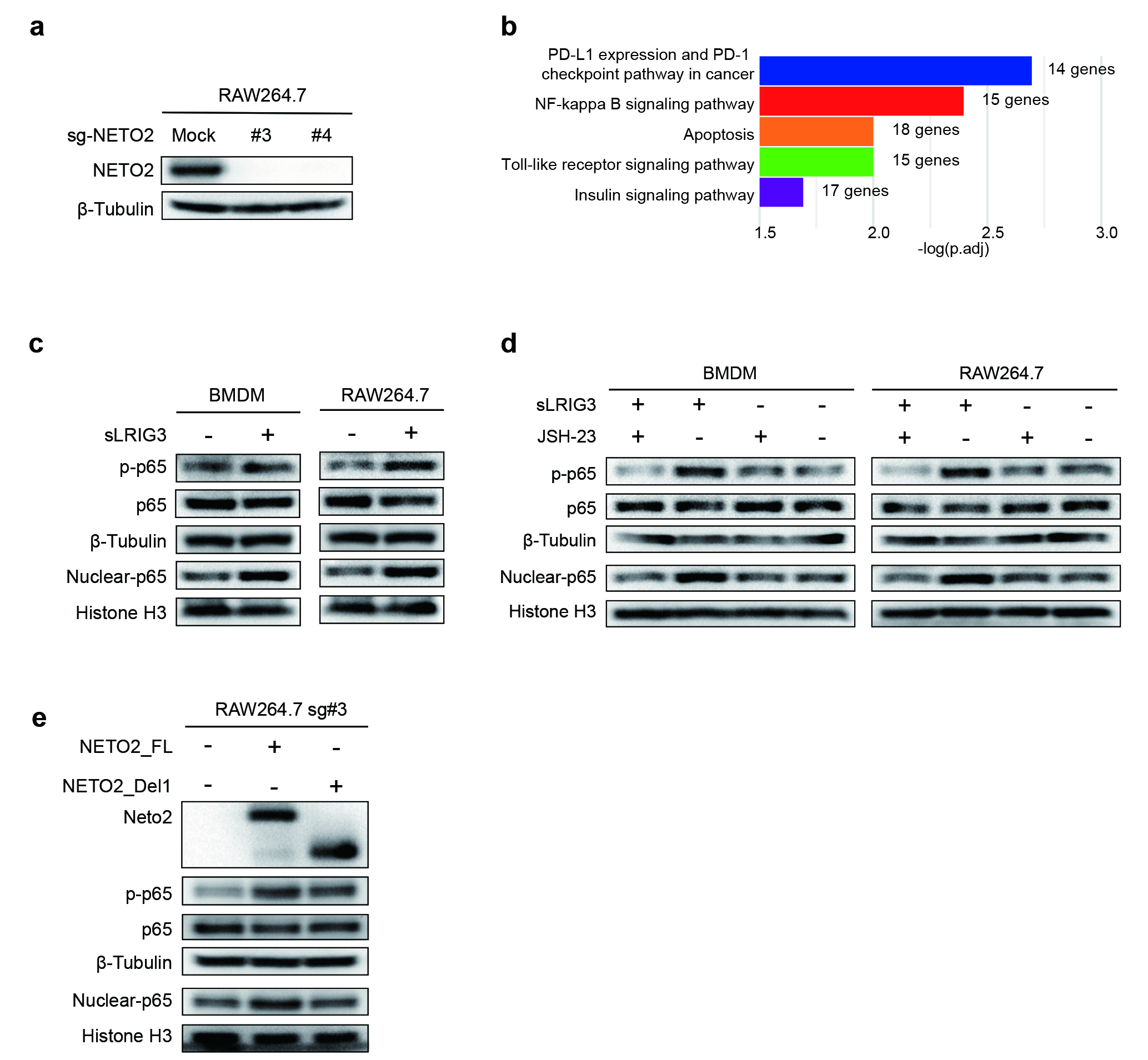

Supplement: Supplementary file 5 — Supplementary figure 4 [file 41419_2023_5555_MOESM5_ESM.tif]

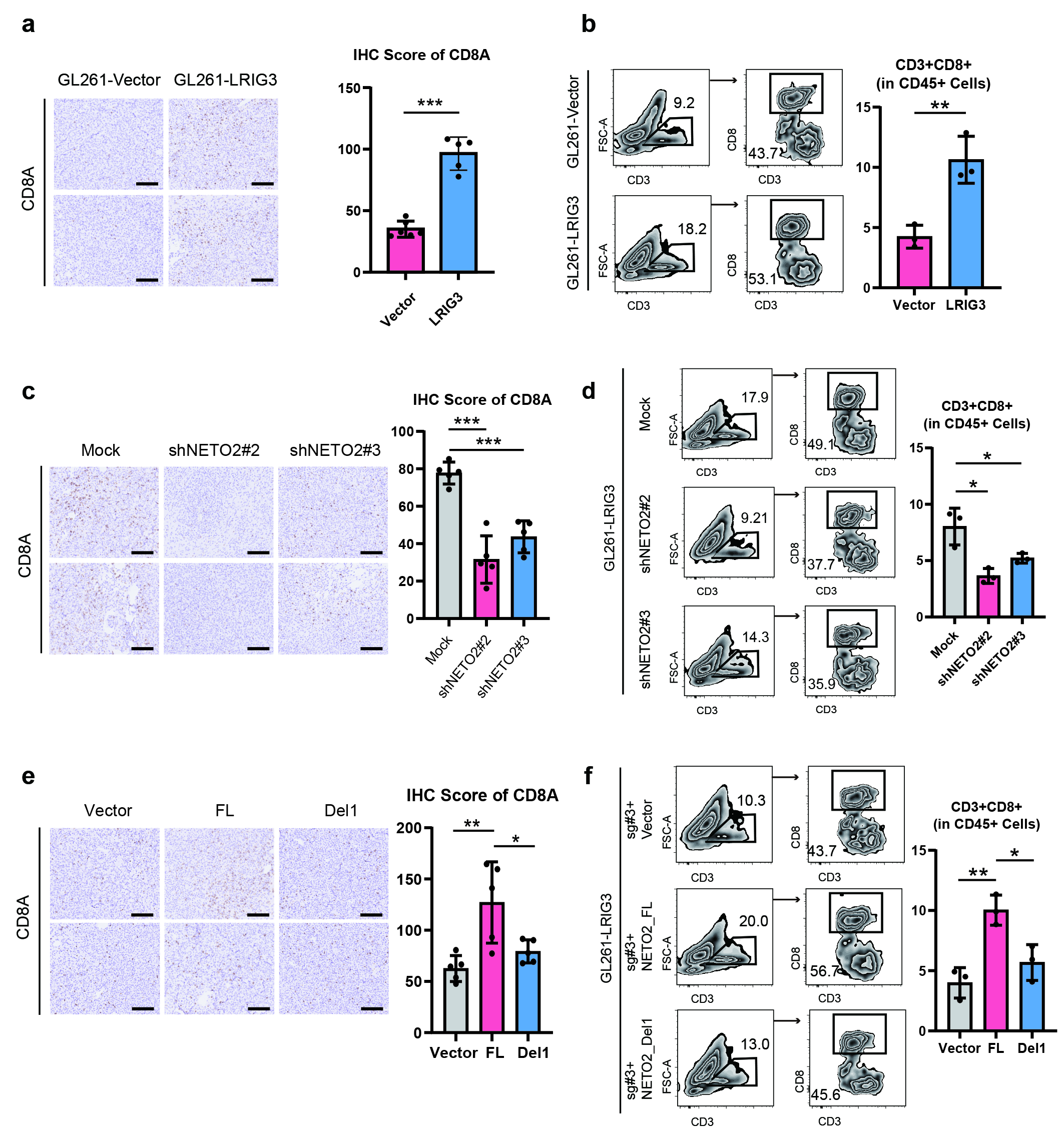

Supplement: Supplementary file 6 — Supplementary figure 5 [file 41419_2023_5555_MOESM6_ESM.tif]

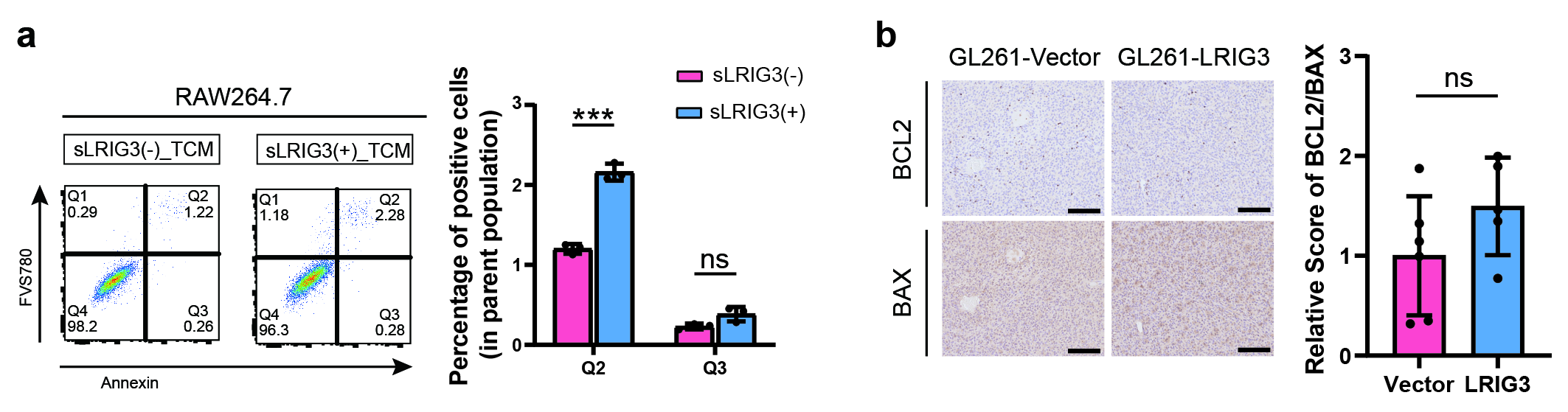

Supplement: Supplementary file 7 — Supplementary figure 6 [file 41419_2023_5555_MOESM7_ESM.tif]

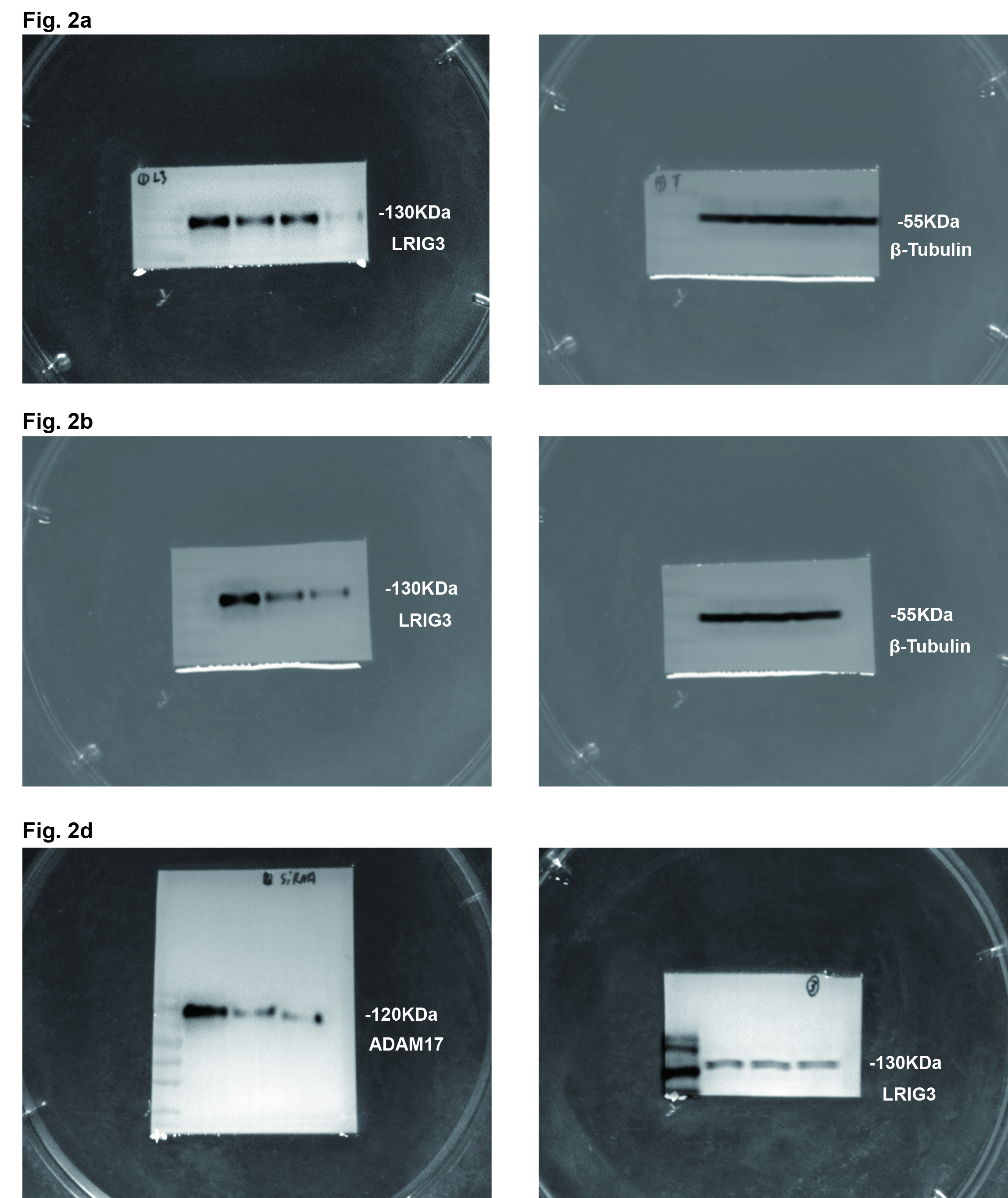

Supplement: Supplementary file 15 — Original Data File of WB bands in figure 2 [file 41419_2023_5555_MOESM15_ESM.tif]

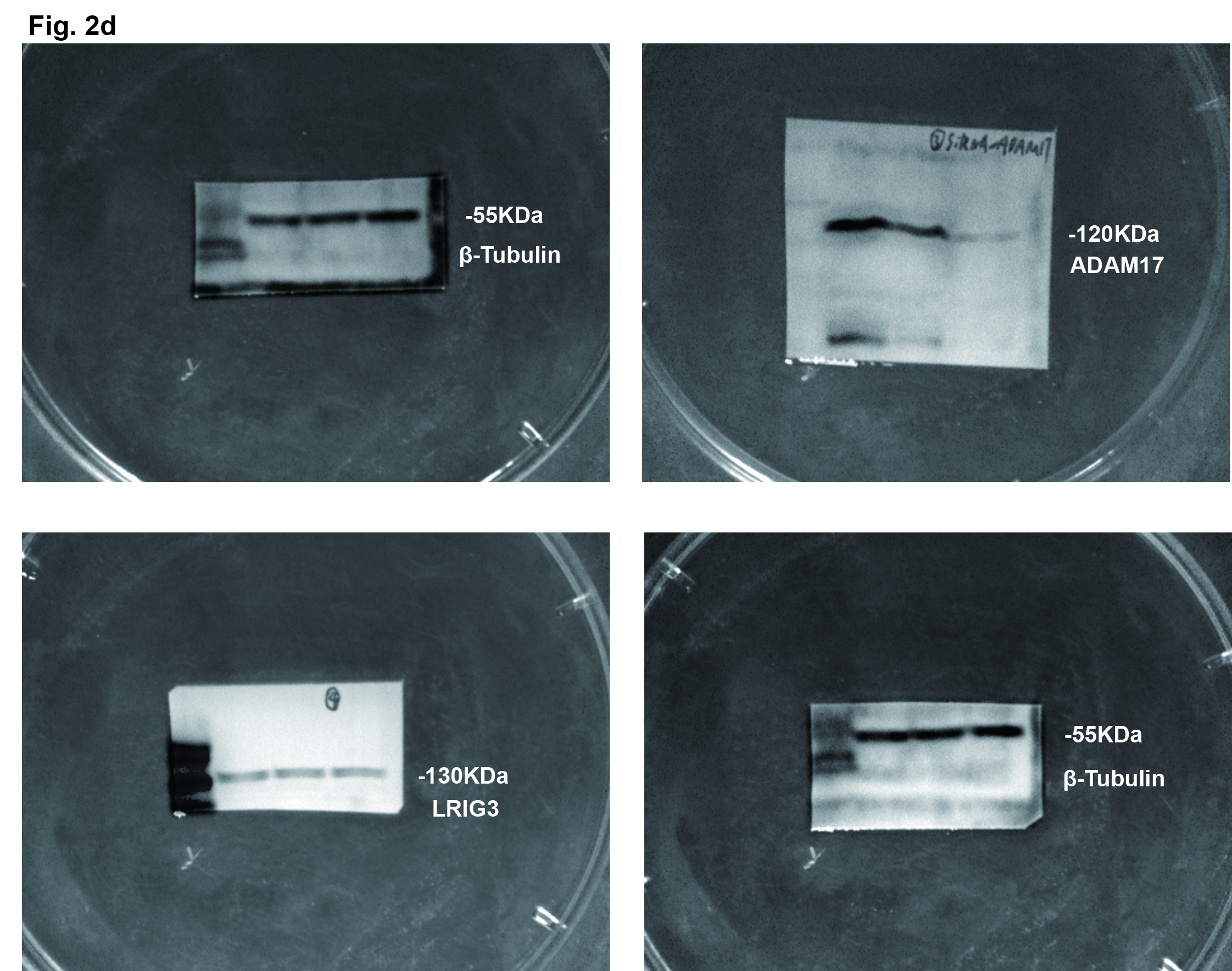

Supplement: Supplementary file 16 — Original Data File of WB bands in figure 2-2 [file 41419_2023_5555_MOESM16_ESM.tif]

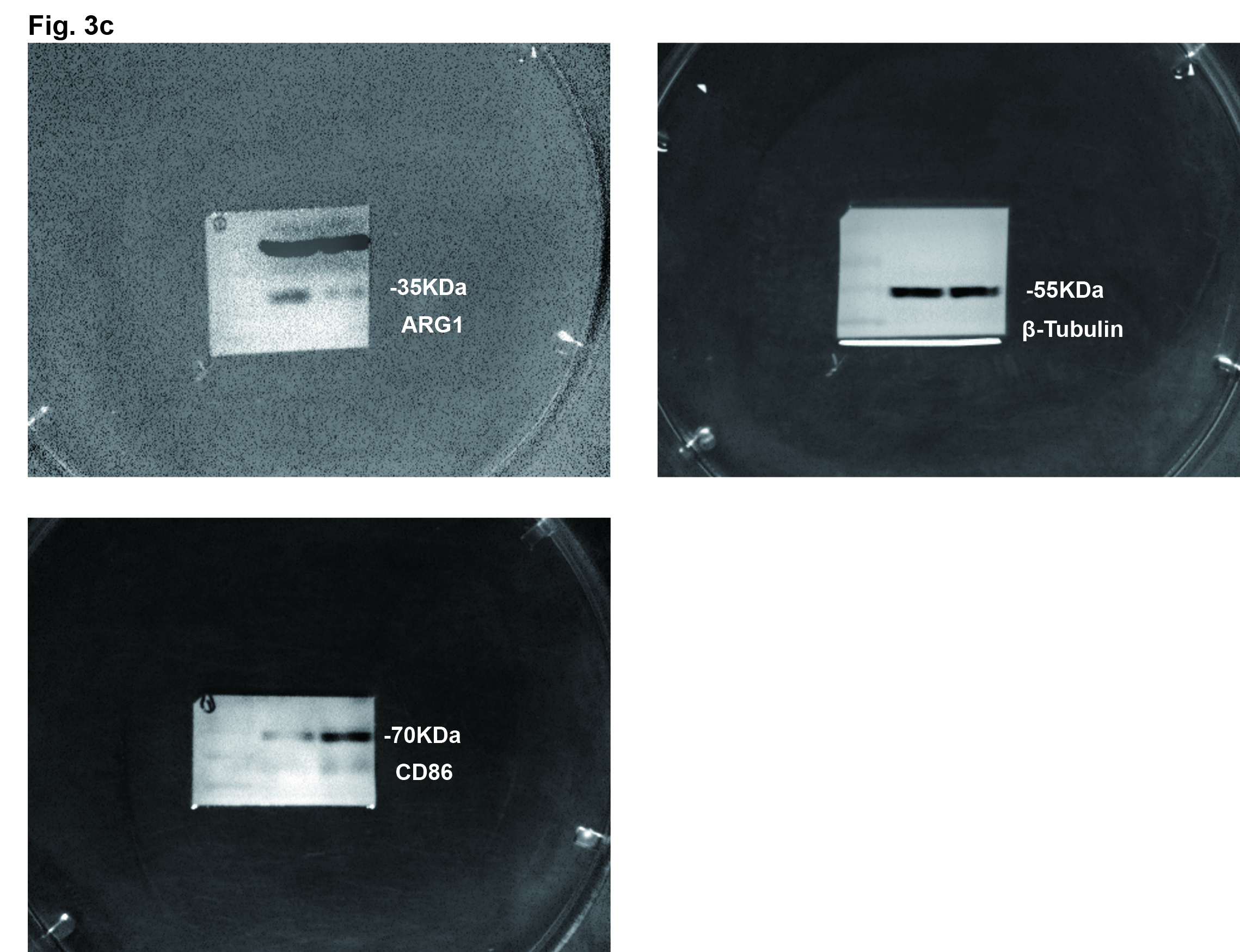

Supplement: Supplementary file 17 — Original Data File of WB bands in figure 3 [file 41419_2023_5555_MOESM17_ESM.tif]

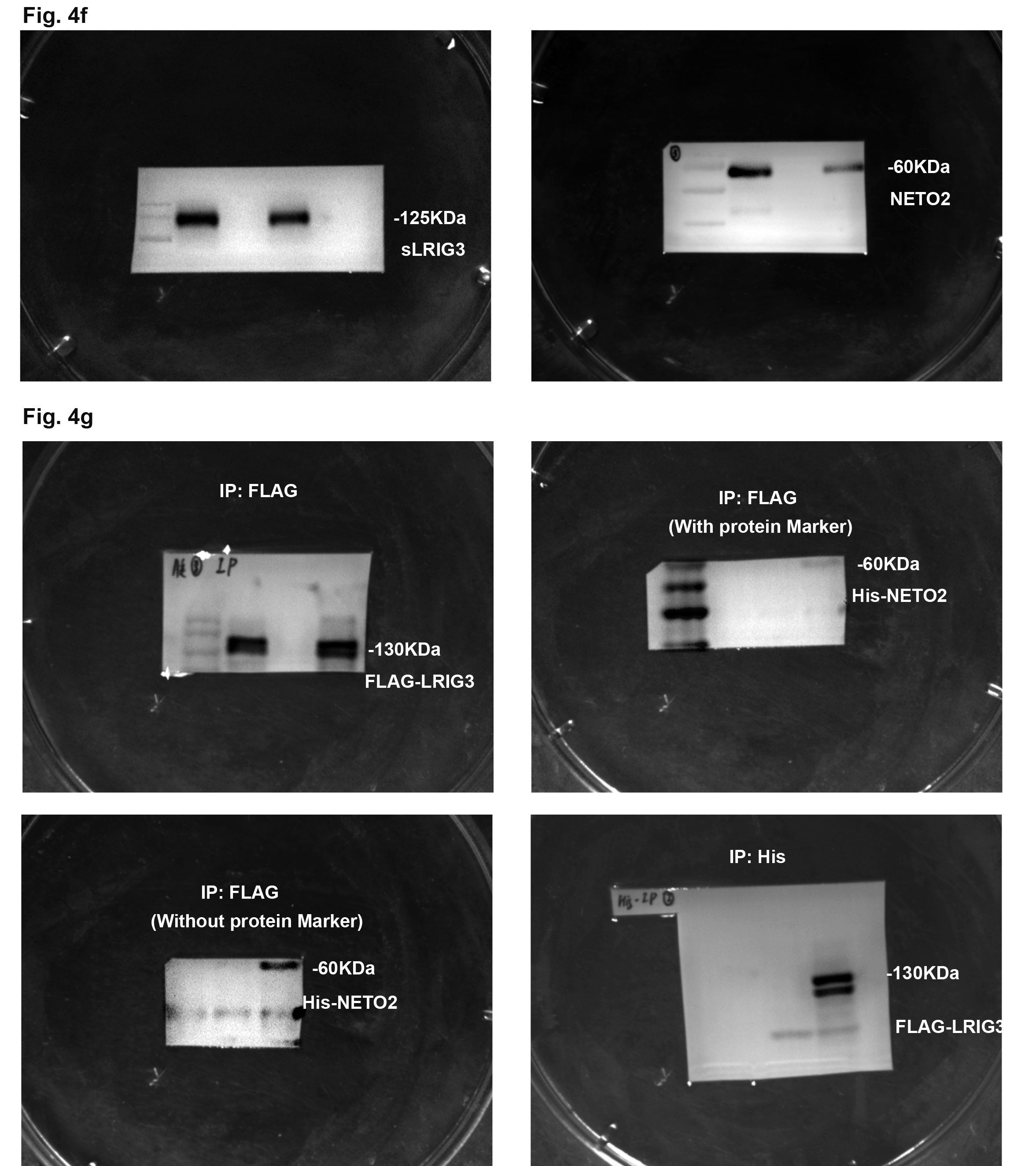

Supplement: Supplementary file 18 — Original Data File of WB bands in figure 4 [file 41419_2023_5555_MOESM18_ESM.tif]

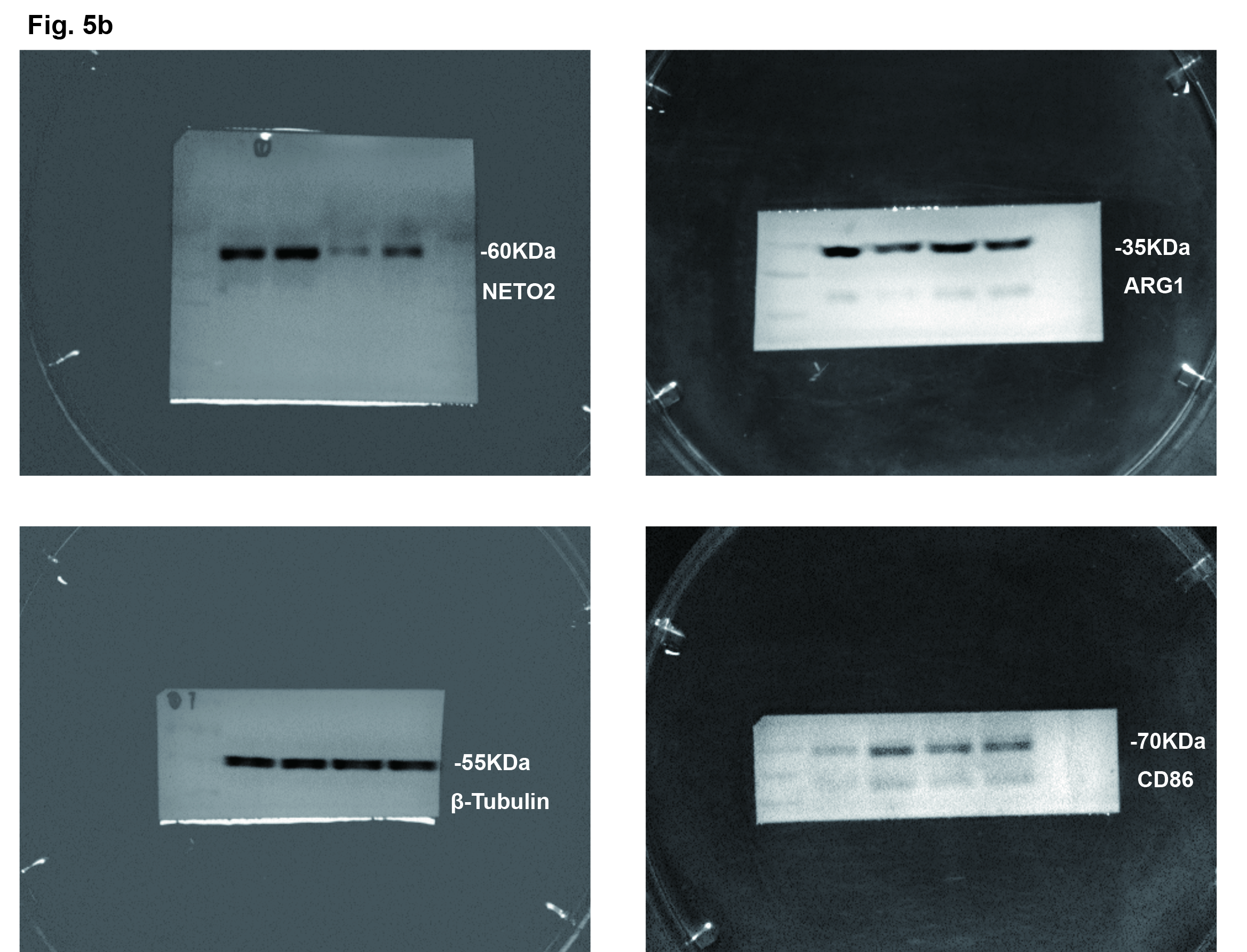

Supplement: Supplementary file 19 — Original Data File of WB bands in figure 5 [file 41419_2023_5555_MOESM19_ESM.tif]

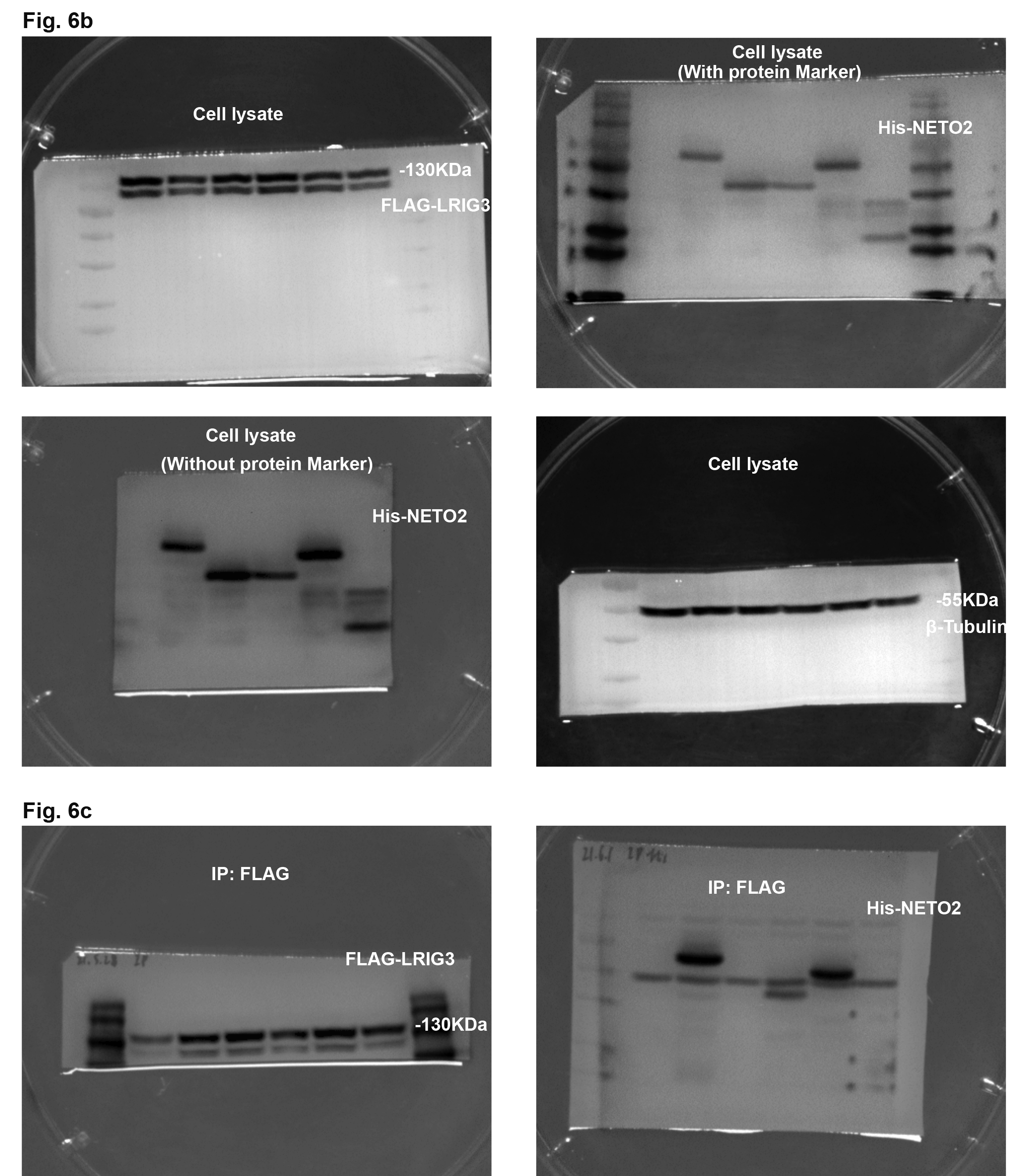

Supplement: Supplementary file 20 — Original Data File of WB bands in figure 6 [file 41419_2023_5555_MOESM20_ESM.tif]

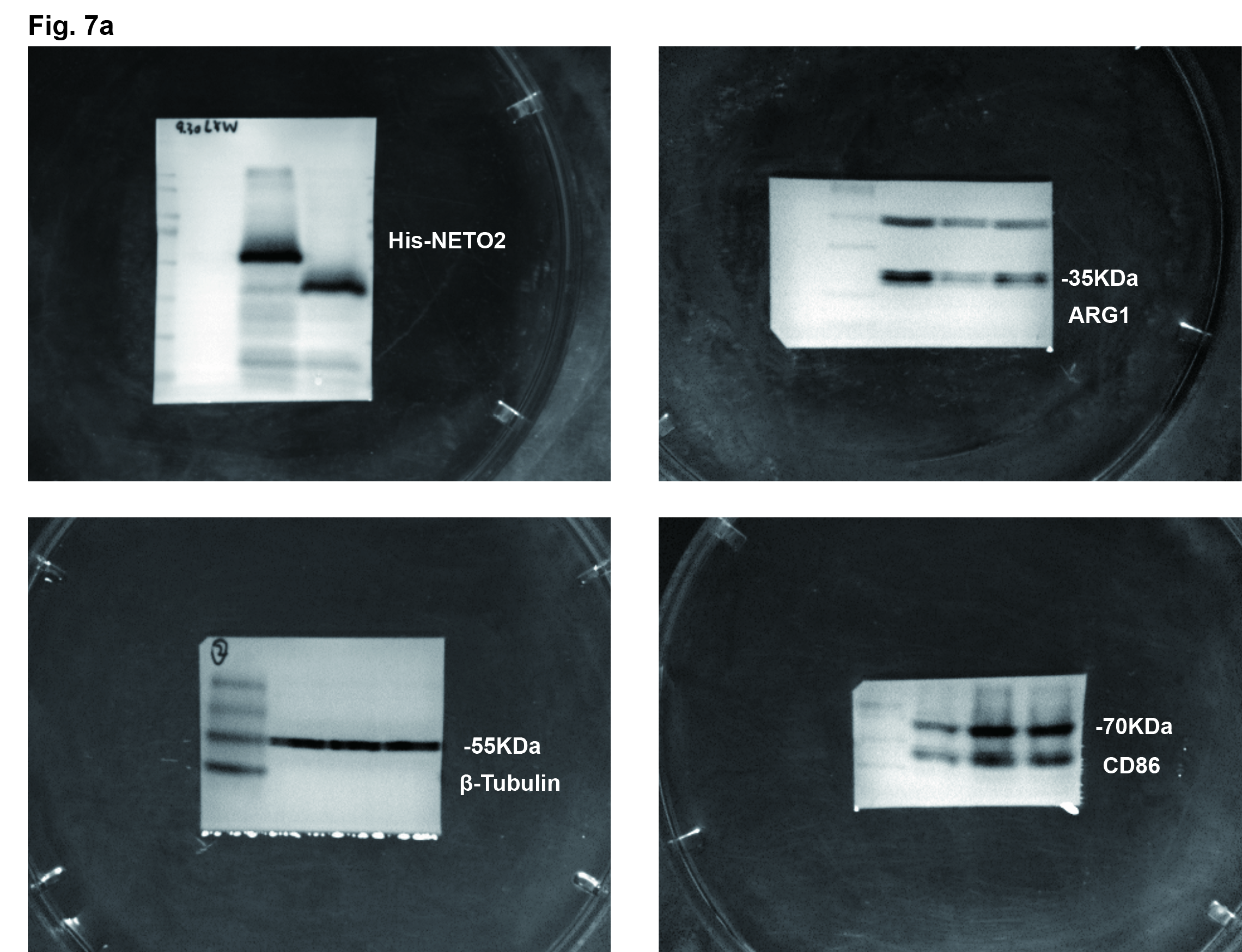

Supplement: Supplementary file 21 — Original Data File of WB bands in figure 7 [file 41419_2023_5555_MOESM21_ESM.tif]

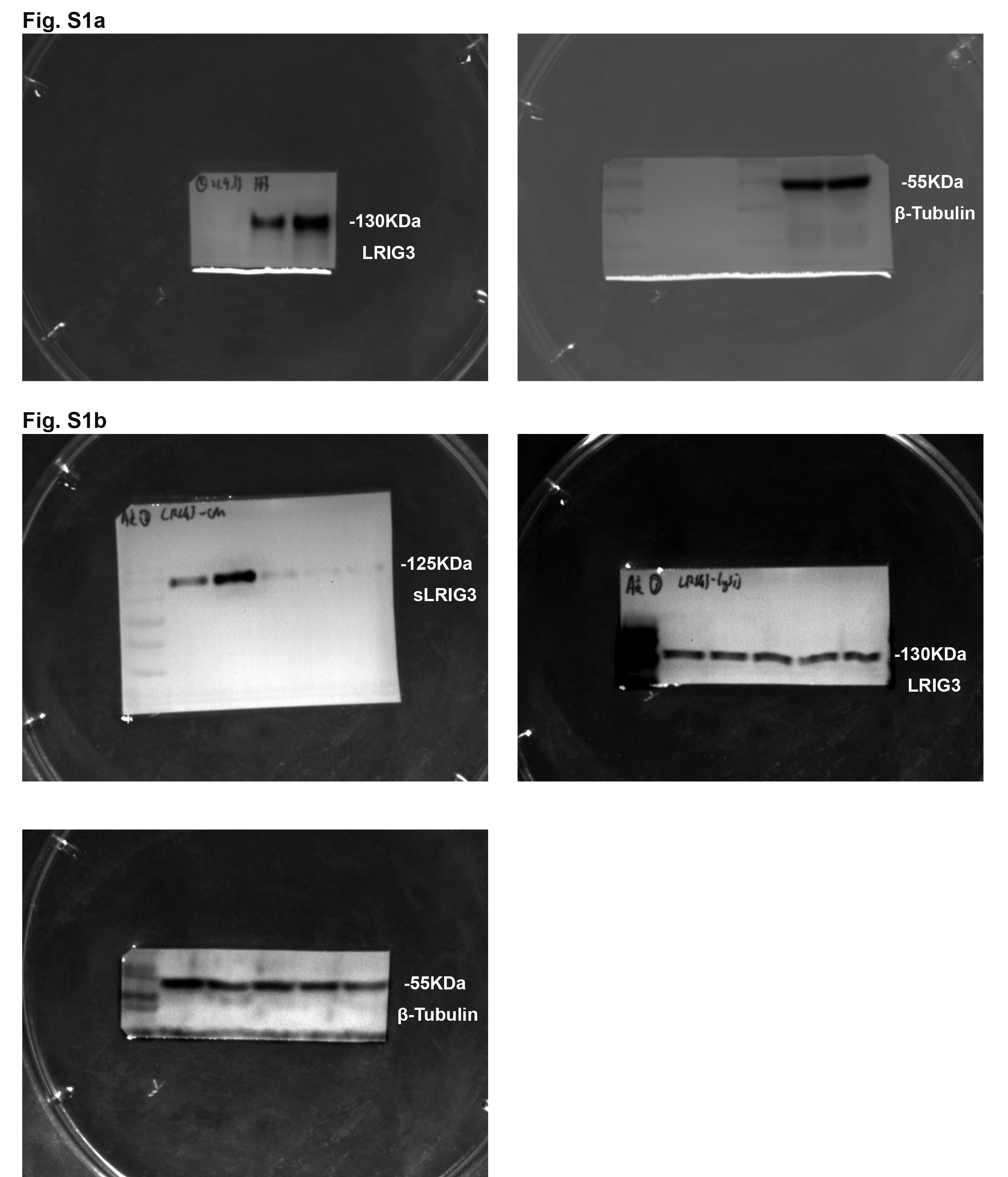

Supplement: Supplementary file 22 — Original Data File of WB bands in supplementary figure 1 [file 41419_2023_5555_MOESM22_ESM.tif]

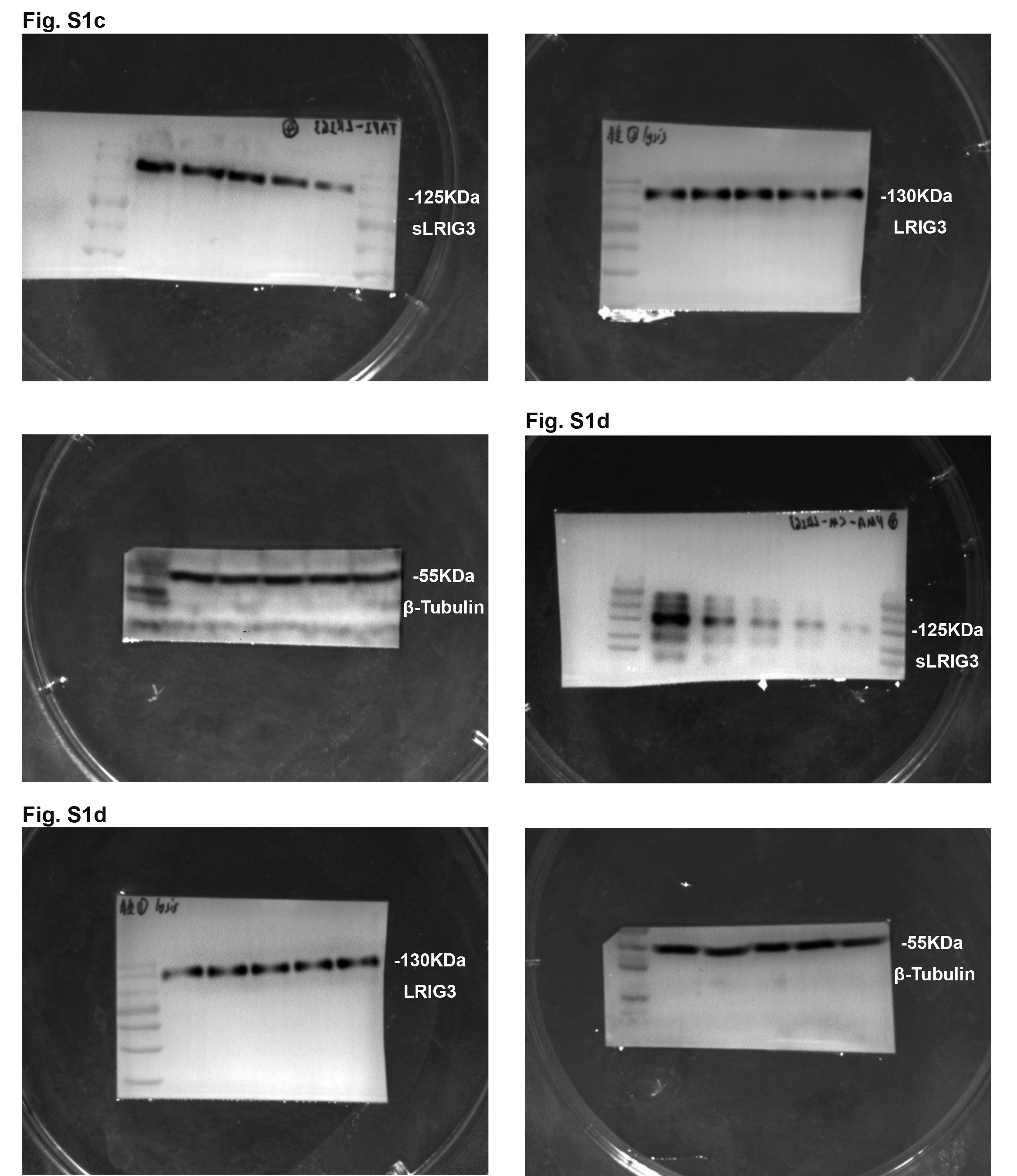

Supplement: Supplementary file 23 — Original Data File of WB bands in supplementary figure 1–2 [file 41419_2023_5555_MOESM23_ESM.tif]

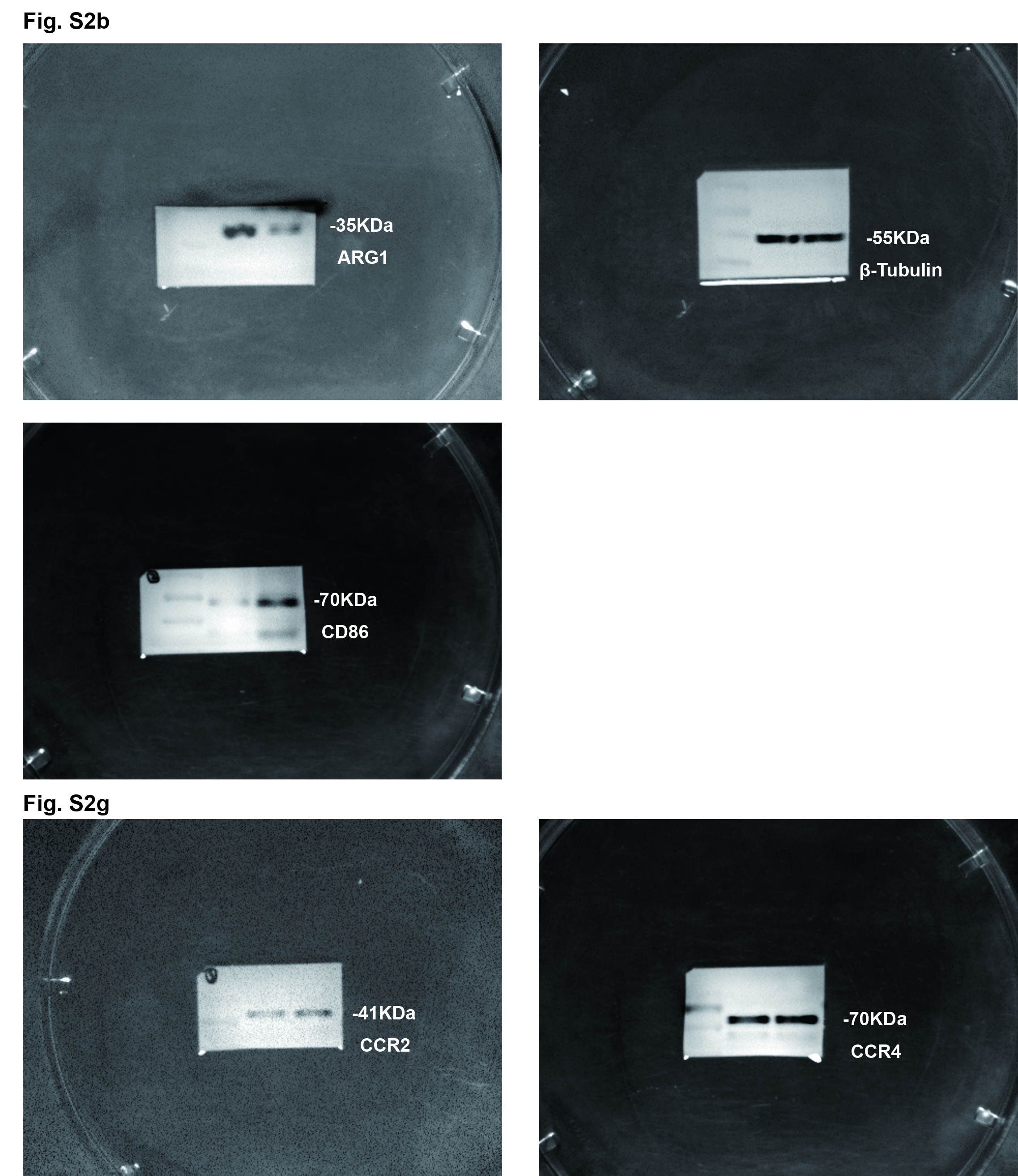

Supplement: Supplementary file 24 — Original Data File of WB bands in supplementary figure 2 [file 41419_2023_5555_MOESM24_ESM.tif]

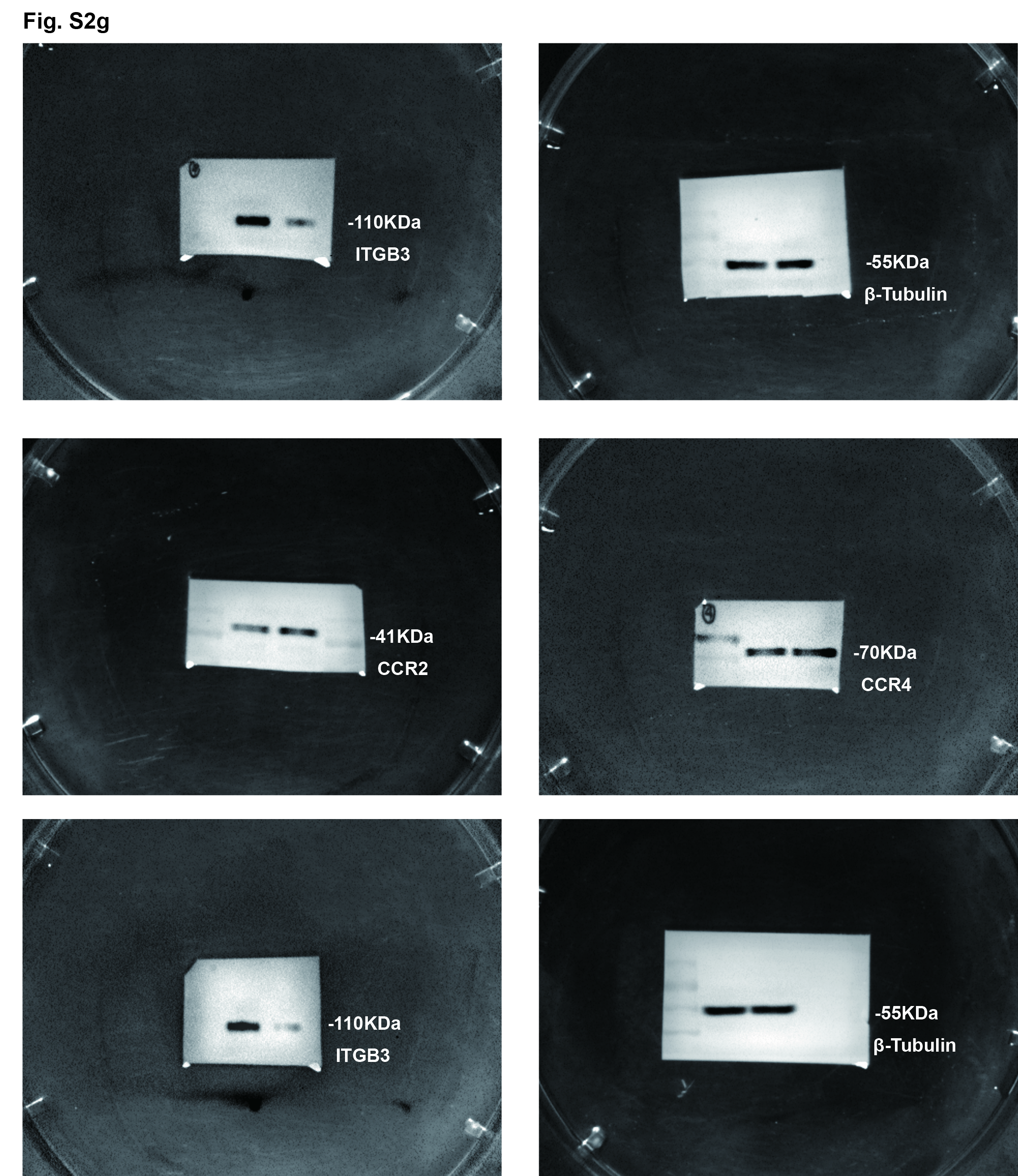

Supplement: Supplementary file 25 — Original Data File of WB bands in supplementary figure 2–2 [file 41419_2023_5555_MOESM25_ESM.tif]

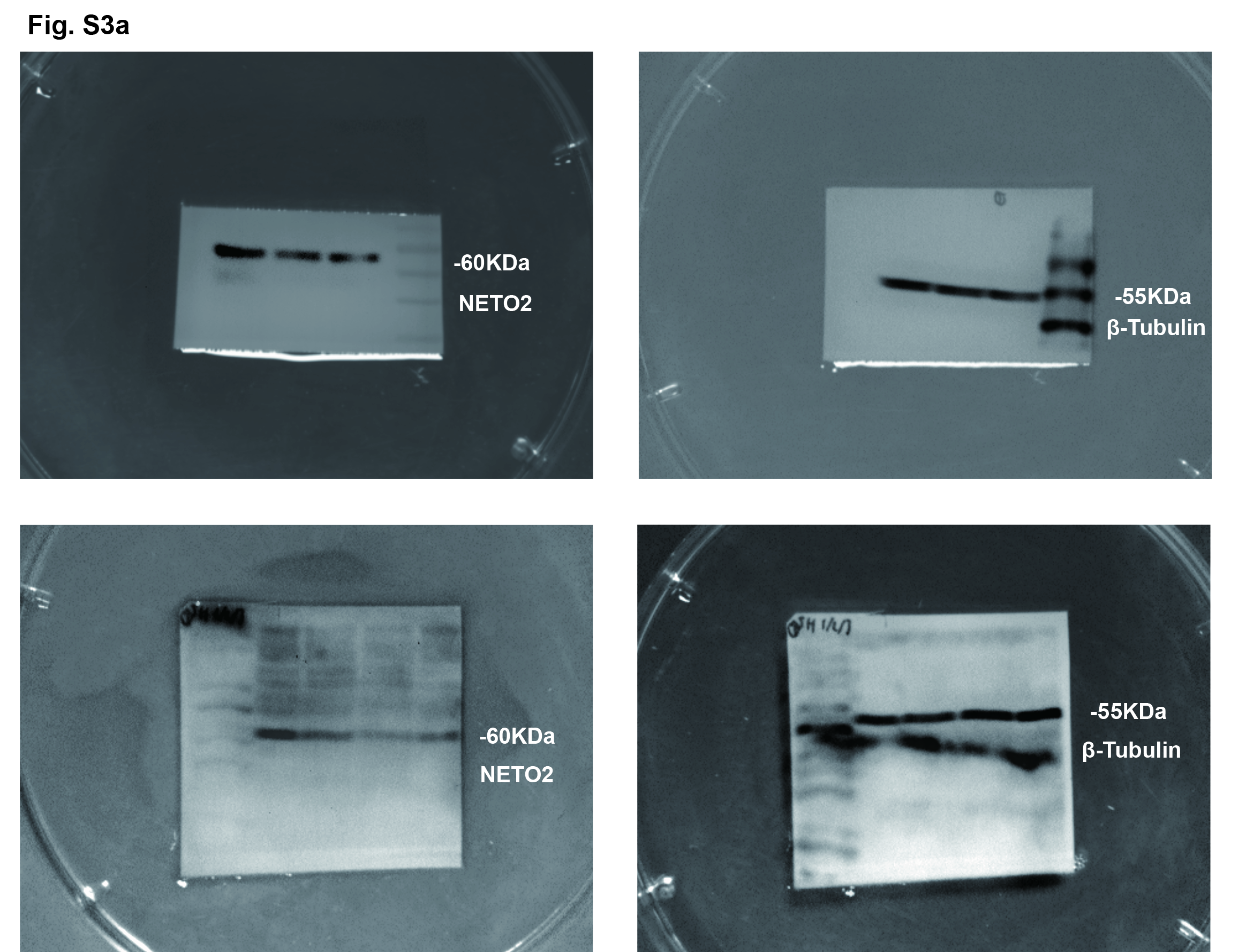

Supplement: Supplementary file 26 — Original Data File of WB bands in supplementary figure 3a [file 41419_2023_5555_MOESM26_ESM.tif]

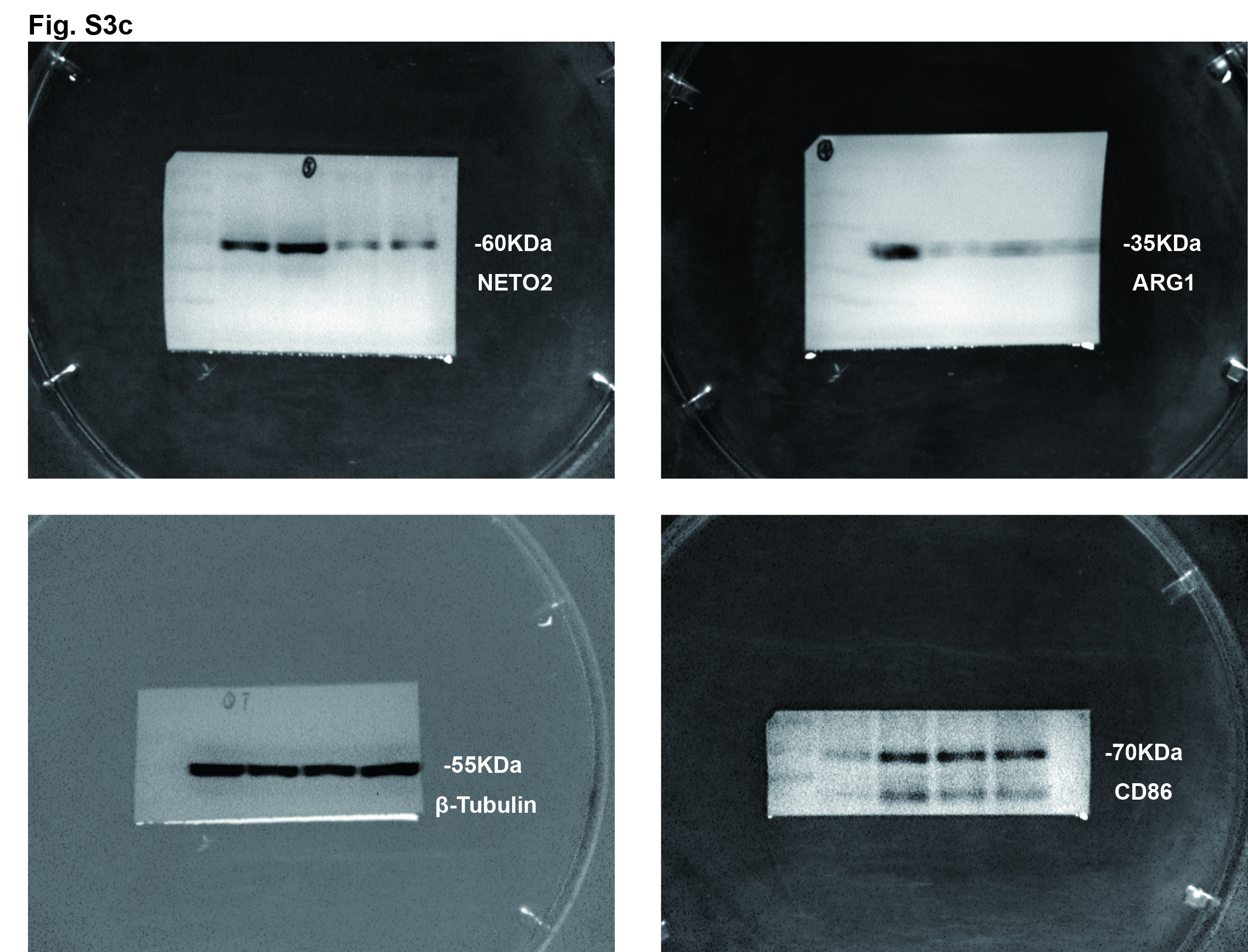

Supplement: Supplementary file 27 — Original Data File of WB bands in supplementary figure c [file 41419_2023_5555_MOESM27_ESM.tif]

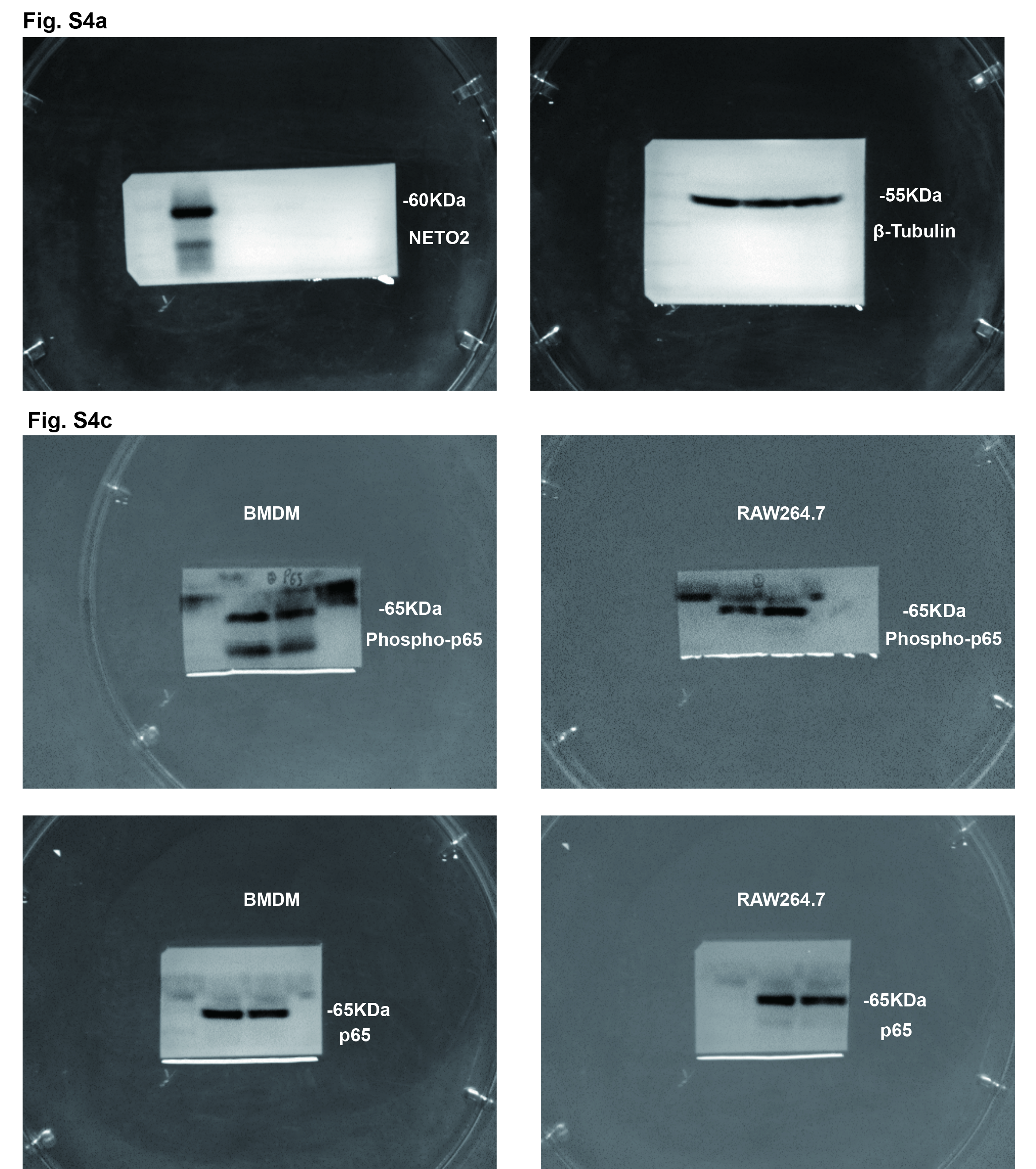

Supplement: Supplementary file 28 — Original Data File of WB bands in supplementary figure 4 [file 41419_2023_5555_MOESM28_ESM.tif]

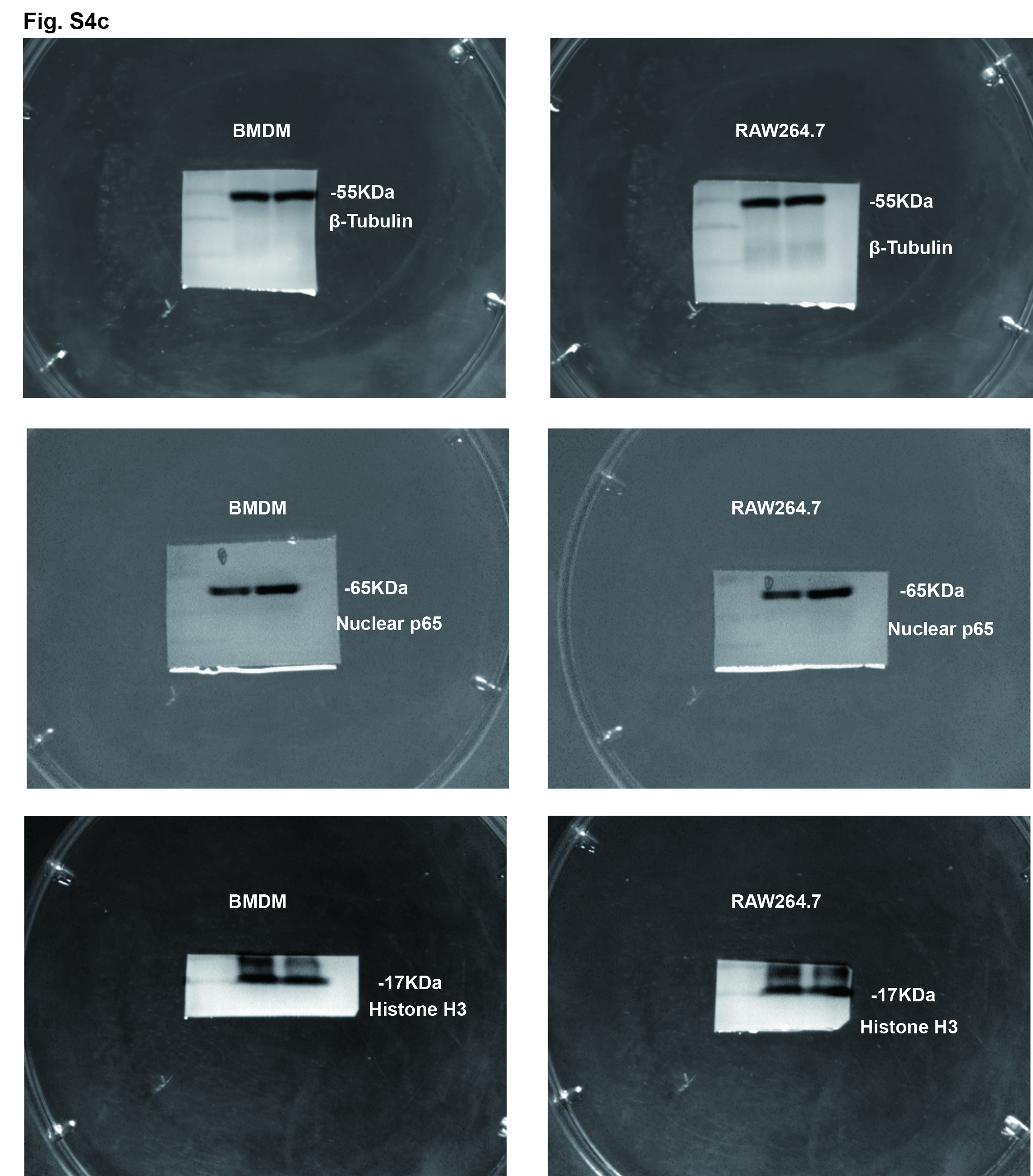

Supplement: Supplementary file 29 — Original Data File of WB bands in supplementary figure 4–2 [file 41419_2023_5555_MOESM29_ESM.tif]

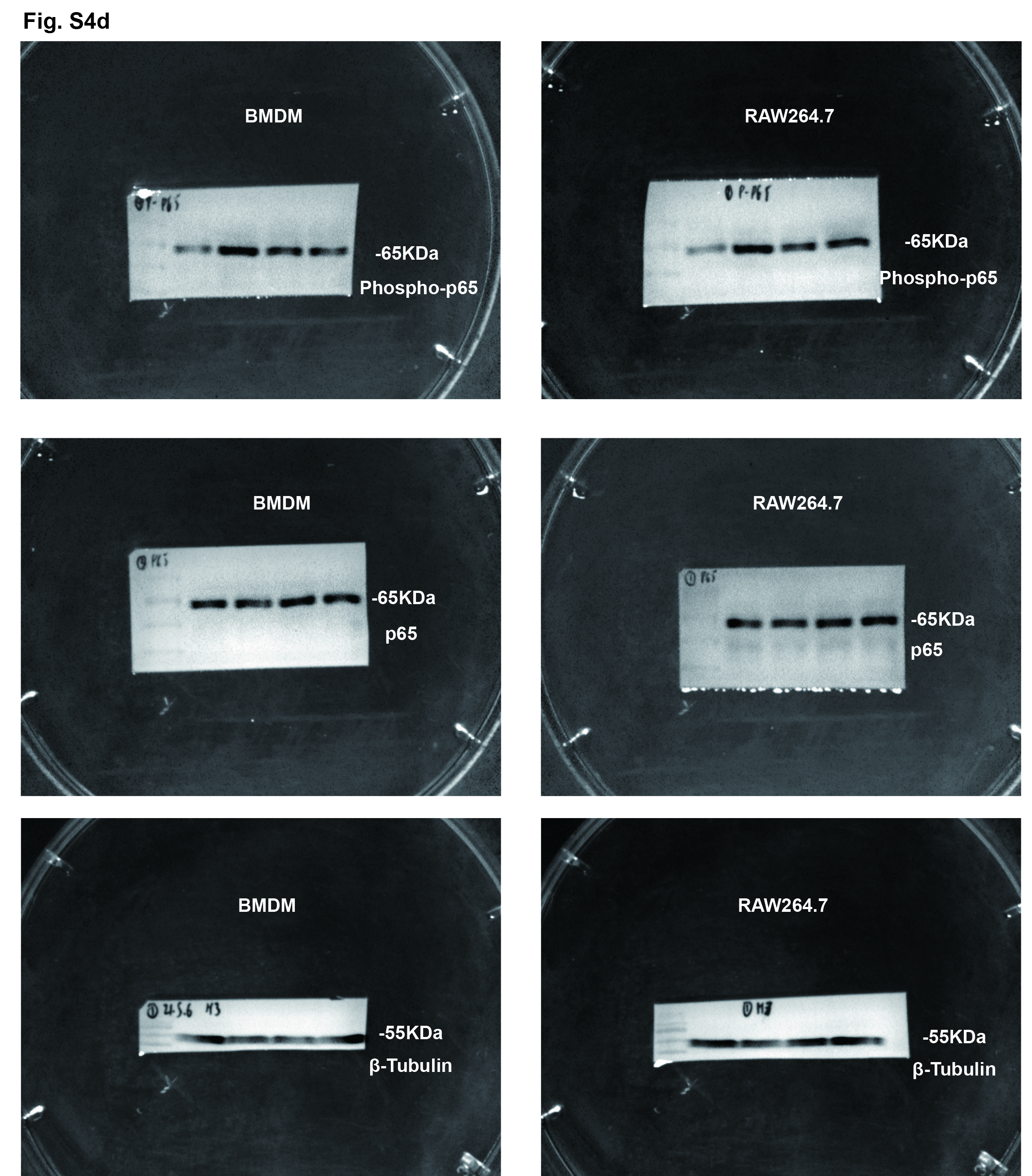

Supplement: Supplementary file 30 — Original Data File of WB bands in supplementary figure 4–3 [file 41419_2023_5555_MOESM30_ESM.tif]

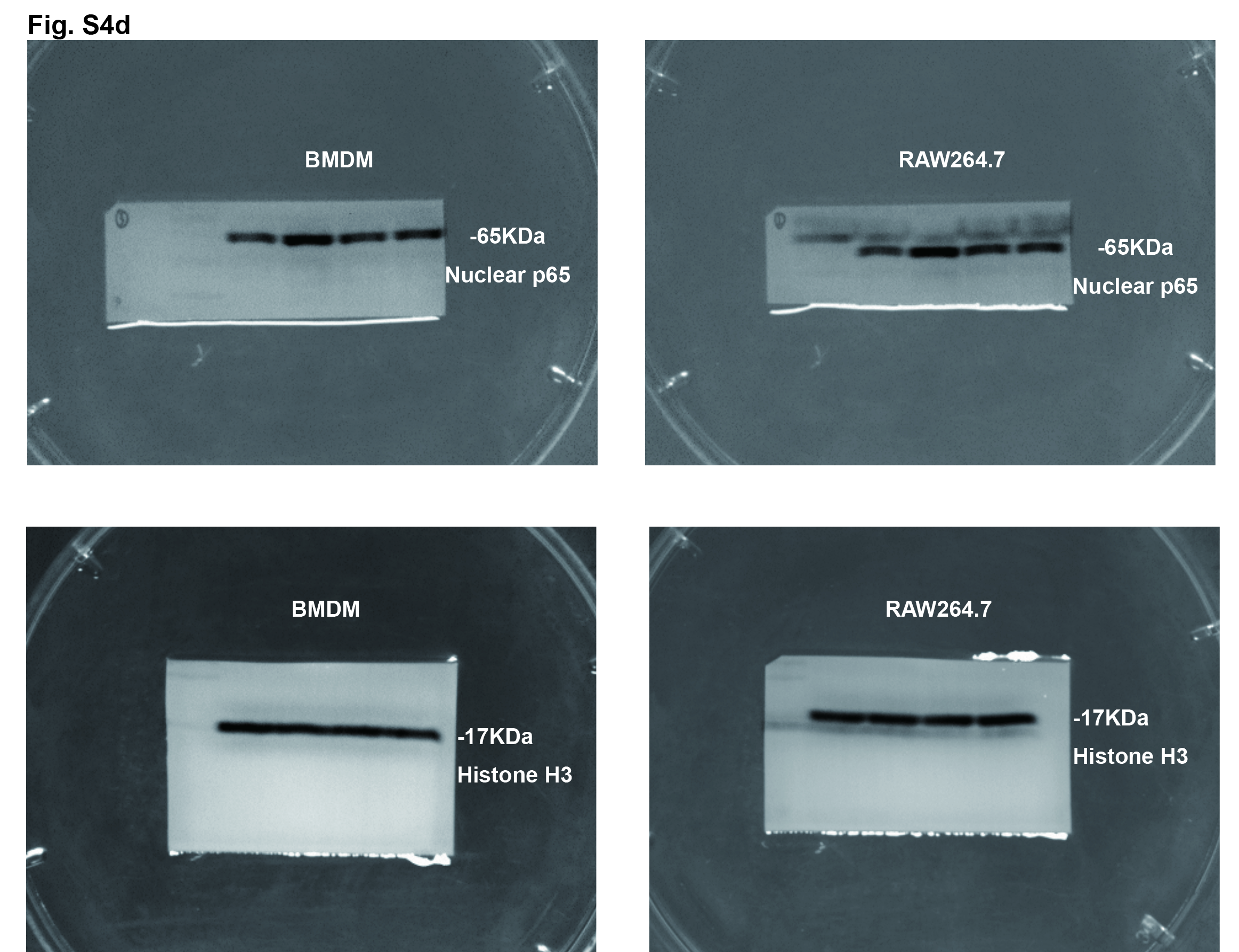

Supplement: Supplementary file 31 — Original Data File of WB bands in supplementary figure 4–4 [file 41419_2023_5555_MOESM31_ESM.tif]

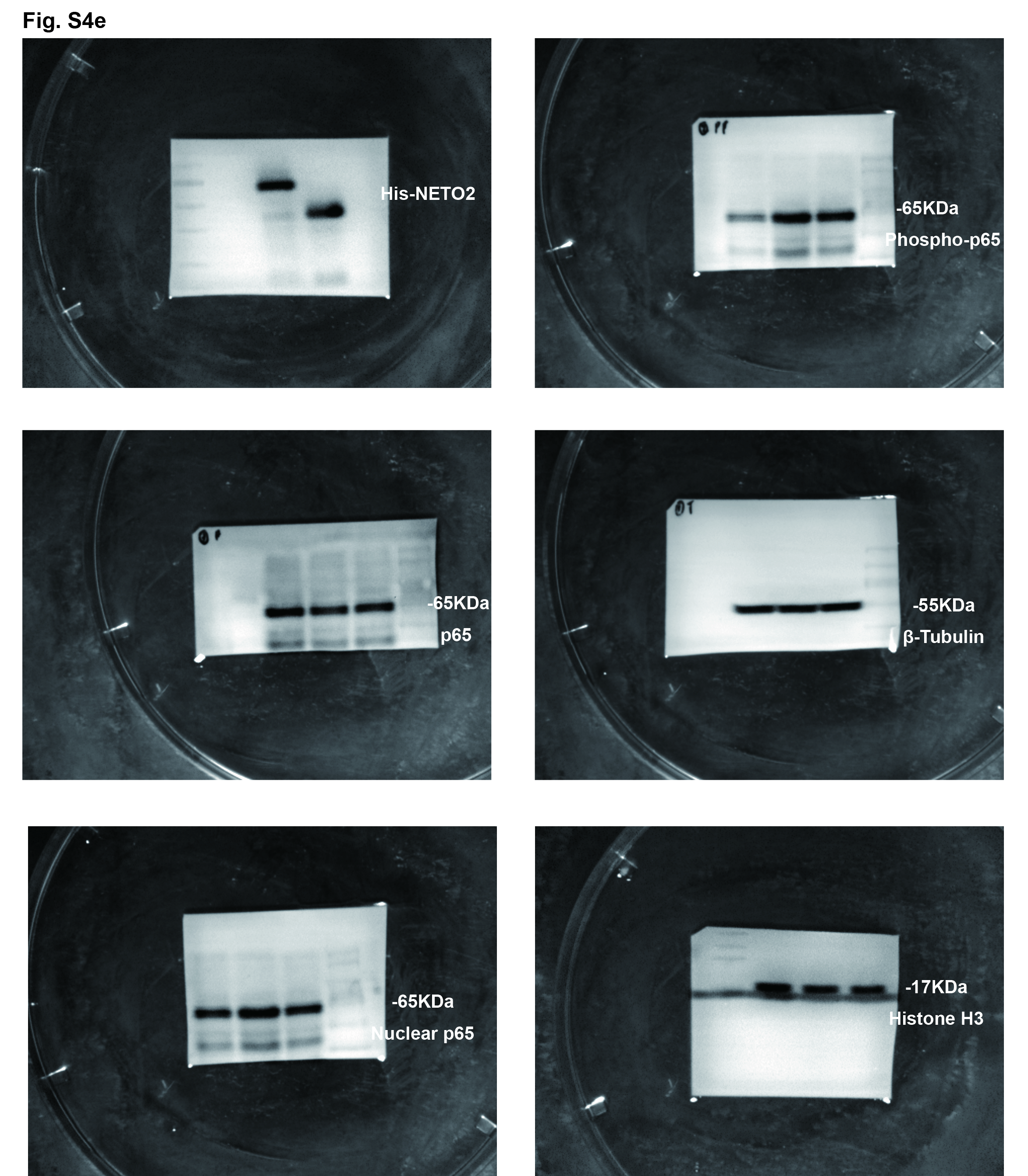

Supplement: Supplementary file 32 — Original Data File of WB bands in supplementary figure 4–5 [file 41419_2023_5555_MOESM32_ESM.tif]
