# Supplementary material for: Glioma-derived LRIG3 interacts with NETO2 in tumor-associated macrophages to modulate microenvironment and suppress tumor growth
Source: Cell Death Dis. 2023 Jan 13;14(1):28. doi: 10.1038/s41419-023-05555-z (PMC9839712; doi:10.1038/s41419-023-05555-z)
Supplement: Supplementary file 8 — Supplementary table 1 [file 41419_2023_5555_MOESM8_ESM.docx]

**Table S1. Clinical features of 71 glioma patients**

| Characteristics |  | Value |
| --- | --- | --- |
| Total sample (n) |  | 71 |
| Age |  |  |
|  | Median (IQR) | 47 (37.5-55) |
|  | < 60 | 58 |
|  | ≥60 | 13 |
| Sex |  |  |
|  | Male | 42 |
|  | Female | 29 |
| Grade |  |  |
|  | II | 21 |
|  | III | 10 |
|  | IV | 40 |

These samples were collected from March 2017 to July 2021.
