# Supplementary material for: Glioma-derived LRIG3 interacts with NETO2 in tumor-associated macrophages to modulate microenvironment and suppress tumor growth
Source: Cell Death Dis. 2023 Jan 13;14(1):28. doi: 10.1038/s41419-023-05555-z (PMC9839712; doi:10.1038/s41419-023-05555-z)
Supplement: Supplementary file 9 — Supplementary table 2 [file 41419_2023_5555_MOESM9_ESM.docx]

**Table S2. A list of target sequences for siRNA/shRNA/sgRNAs**

| Prioject | Order | Target sequence |
| --- | --- | --- |
| **SiRNA/shRNA** |  |  |
| *ADAM17* |  |  |
|  | #1 | CCAGCAGCATTCGGTAAGAAA |
|  | #2 | CCTATGTCGATGCTGAACAAA |
| *Adam17* |  |  |
|  | #1 | GGCTACATTTCAGGCACTCGG |
|  | #2 | GGCTAGAACCCTAGAGTCAGG |
| *Neto2* |  |  |
|  | #1 | GCTATTATATAGAGCCATCAT |
|  | #2 | GCATCCATATCCATCGACTTT |
|  | #3 | CGGGAAGATTCATGTGGATTA |
|  |  |  |
| **SgRNA** |  |  |
| *Neto2* |  |  |
|  | #1 | GTCAGCTCTATTCGTTGACG |
|  | #2 | TCAAACCGACATTCGAATGA |
|  | #3 | CCGTTGACCTAATTAATGCA |
|  | #4 | CCTCTTATAGATCGTTACTG |
