# Supplementary material for: Glioma-derived LRIG3 interacts with NETO2 in tumor-associated macrophages to modulate microenvironment and suppress tumor growth
Source: Cell Death Dis. 2023 Jan 13;14(1):28. doi: 10.1038/s41419-023-05555-z (PMC9839712; doi:10.1038/s41419-023-05555-z)
Supplement: Supplementary file 10 — Supplementary table 3 [file 41419_2023_5555_MOESM10_ESM.docx]

**Table S3. A list of primers used for RT­qPCR assay**

| Gene name |  | Oligo Sequence (5'-3') |
| --- | --- | --- |
| *Tnf* | Forward | GACGTGGAACTGGCAGAAGAG |
|  | Reverse | TTGGTGGTTTGTGAGTGTGAG |
| *Il1b* | Forward | GCAACTGTTCCTGAACTCAACT |
|  | Reverse | ATCTTTTGGGGTCCGTCAACT |
| *Nos2* | Forward | GTTCTCAGCCCAACAATACAAGA |
|  | Reverse | GTGGACGGGTCGATGTCAC |
| *Arg1* | Forward | CTCCAAGCCAAAGTCCTTAGAG |
|  | Reverse | AGGAGCTGTCATTAGGGACATC |
| *Cd301* | Forward | TGAGAAAGGCTTTAAGAACTGGG |
|  | Reverse | GACCACCTGTAGTGATGTGGG |
| *Mrc1* | Forward | CTCTGTTCAGCTATTGGACGC |
|  | Reverse | CGGAATTTCTGGGATTCAGCTTC |
| *Gapdh* | Forward | AGGTCGGTGTGAACGGATTTG |
|  | Reverse | TGTAGACCATGTAGTTGAGGTCA |
