# Supplementary material for: Glioma-derived LRIG3 interacts with NETO2 in tumor-associated macrophages to modulate microenvironment and suppress tumor growth
Source: Cell Death Dis. 2023 Jan 13;14(1):28. doi: 10.1038/s41419-023-05555-z (PMC9839712; doi:10.1038/s41419-023-05555-z)
Supplement: Supplementary file 13 — Author Contribution Statement [file 41419_2023_5555_MOESM13_ESM.docx]

GD (the corresponding author) designed and supervised the study. LY (the first author) and WW(the co-first author) conducted most of the experiments. HW, HX and MF participated in the acquirement of experimental data. DQ and MF provided the technical support. HY and WBF acquared the clinical data associated with the study. LY wrote the draft, MF (the co-corresponding author) and GD revised it. All authors read and approved the final version of the manuscript.
